# Unveiling the Enzymatic Degradation Process of Biobased Thiophene Polyesters

**DOI:** 10.3389/fchem.2021.771612

**Published:** 2021-11-15

**Authors:** Federico A. Bertolini, Michelina Soccio, Simone Weinberger, Giulia Guidotti, Massimo Gazzano, Georg M. Guebitz, Nadia Lotti, Alessandro Pellis

**Affiliations:** ^1^ Department of Agrobiotechnology, Institute of Environmental Biotechnology, University of Natural Resources and Life Sciences, Vienna, Tulln an der Donau, Austria; ^2^ Department of Civil, Chemical, Environmental and Materials Engineering, University of Bologna, Bologna, Italy; ^3^ Organic Synthesis and Photoreactivity Institute, CNR, Bologna, Italy; ^4^ Austrian Centre of Industrial Biotechnology, Tulln an der Donau, Austria; ^5^ Core Facility Bioactive Molecules Screening and Analysis, University of Natural Resources and Life Sciences, Vienna, Tulln an der Donau, Austria

**Keywords:** polyesters, thiophene-based polymers, cutinases, circular economy, biodegradability, circular materials

## Abstract

In the past 20 years, scientific research focused on the identification of valid alternatives to materials of fossil origin, in particular, related to biobased polymers. Recently, the efforts led to the synthesis of thiophene-based polymers (TBPs), a new class of polyesters based on 2,5-thiophenedicarboxylic acid (TPCA) that can be industrially produced using biomass-derived molecules. In this study, TBPs were synthesized using diols with different chain length (from C4 to C6) leading to poly(butylene 2,5-thiophenedicarboxylate) (PBTF), poly(pentamethylene 2,5-thiophenedicarboxylate) (PPeTF), and poly(hexamethylene 2,5-thiophenedicarboxylate) (PHTF), respectively, that were processed to thin films. To investigate enzymatic hydrolysis of these polymer films, cutinase 1 (Thc_cut1) and cutinase 2 (Thc_cut2) from *Thermobifida cellulosilytica* were recombinantly expressed in the host *E. coli* and purified. After 72 h of incubation at 65°C with 5 µM Thc_cut1, weight loss and HPLC analysis indicated 9, 100, and 80% degradation of PBTF, PPeTF, and PHTG with a concomitant release of 0.12, 2.70, and 0.67 mM of TPCA. The SEM analysis showed that tiny holes were formed on the surface of the films and after 72 h PPeTF was completely degraded. The LC-TOF/MS analysis indicated that Thc_cut2 in particular released various oligomers from the polymer during the reaction. In addition, the FTIR analysis showed the formation of novel acid and hydroxyl groups on the polymer surfaces. The results showed that the two used thermostable cutinases are promising biocatalysts for the environmentally friendly degradation of TPCA-based polyesters, in view of a possible sustainable recycling of plastic waste through resynthesis processes.

## 1 Introduction

Global plastics production is continuously increasing, and in 2018, it reached 359 million metric tons, of which 61.8 million metric tons are produced in Europe alone. The public’s awareness of the problems caused by pollution due to plastics dispersed in the environment has been increasingly growing in recent years. In the ocean, the presence of the great pacific garbage patch is well known (Lebreton et al., 2018), while recently, evidence has emerged that debris are also accumulating in the deep layers of water (Egger et al., 2020). A problem related to human nutrition also emerged as the presence of plastics was found in the fish caught in the North Sea, a region with high fish stocks (Foekema et al., 2013). The current situation and the need for implementing a plastic’s circular economy are pushing the development of eco-friendly plastics that are both biobased and biodegradable, being capable of replacing those currently in use. This is especially important for applications where a short product life is expected and contamination prevents recycling such as packaging, consumer products, cosmetics, and healthcare.

The pandemic caused by COVID-19 still afflicting the planet resulted in an increased utilization and disposal of single-use plastics, as proved by the increased demand of plastics by 40% in packaging and 17% in other applications, including medical uses. Indeed, safety concerns push consumers to prefer fresh-food packaged in plastic containers to avoid food contamination and to extend the shelf-life.

In this scenario, biodegradable and biobased plastics are the preferred options to efficiently manage eco-friendly plastics waste. On the one side, total biodegradation of waste reduces its volume and the consequent environmental impact; on the other side, a controlled enzymatic biodegradation with the production of the monomer used to produce the starting plastics could represent a valid and more sustainable and specific alternative to chemical recycling, especially for blended materials and mixed wastes.

In the packaging field, the most commonly used plastics are polyolefines (polyethylene and polypropylene) and poly(ethylene terephthalate) (PET), the latter to produce water and soft drink bottles. New routes have been developed for the production of biobased terephthalic acid (TA) (Pellis et al., 2016), but nowadays PET is still almost entirely produced from fossil sources. Research efforts are therefore focusing on replacing TA with compounds obtained from renewable sources. An important step forward in this direction has been made, thanks to the identification of 2,5-furandicarboxylic acid (FDCA) as a potential substitute for TA (de Jong et al., 2012), since large-scale production from renewable sources was recently demonstrated (Kim et al., 2018). Starting from PET and replacing TA with FDCA, poly(ethylene 2,5-furanoate) (PEF) was produced, as well as other polymers containing FDCA and diols with varying carbon chain length (from 2 to 12 carbon atoms) (Papageorgiou et al., 2016; Arnaud et al., 2017; Guidotti et al., 2020).

Therefore, FDCA-based polymers, such as PEF, have a biobased origin and show excellent results in terms of gas permeability (Burgess et al., 2015), in particular O_2_-barrier properties (Burgess et al., 2014) and mechanical characteristics (Guidotti et al., 2020). In addition to these features, recent studies have shown that FDCA-based polymers have also a better enzymatic degradability than the TA-based ones (Gamerith et al., 2017; Guidotti et al., 2020).

Recently, a new class of polymers has been synthesized in which the FDCA is replaced by the biobased 2,5-thiophenedicarboxylic acid (TPCA) (Weinberger et al., 2017; Guidotti et al., 2018d), in order to obtain high performance polymers. It has been observed that the gas-barrier properties of TPCA-based polymers can be comparable or even better than those of FDCA-based polymers (Weinberger et al., 2017; Guidotti et al., 2018b; Guidotti et al., 2018c; Guidotti et al., 2018d). Among the TPCA-based polymers, the most interesting one is poly(butylene 2,5-thiophenedicarboxylate) (PBTF) since it shows excellent gas-barrier properties and similar enzymatic degradability when compared to poly(butylene furanoate) (Weinberger et al., 2017; Guidotti et al., 2018a).

However, the impact of the diol chain length of TPCA-based polymers on enzymatic hydrolysis has not yet been investigated. Therefore, in this work, two hydrolytic enzymes from *Thermobifida cellulosilytica*, namely, cutinase 1 (Thc_cut1) and cutinase 2 (Thc_cut2), were used to investigate the hydrolysis of poly(pentanediol 2,5-thiophenedicarboxylate) (PPeTF) and poly(hexanediol 2,5-thiophenedicarboxylate) (PHTF) compared to PBTF.

## 2 Materials and Methods

### 2.1 Polymer Synthesis

Prior to high molecular weight polymers’ synthesis, esterification of 2,5-thiophenedicarboxylic acid was carried out, according to the procedure already described (Guidotti et al., 2018b). In addition, the diacid and a large excess of anhydrous methanol (1:30 mol) were heated to 70°C under nitrogen flux until complete dissolution of the diacid. After that, the mixture was cooled to room temperature, and thionyl chloride, in the same molar amount as the diacid, was added by means of a drip funnel. Then, the solution was reheated to 70°C and kept 3 h under stirring in inert atmosphere before being quenched in an ice-cold water bath. The so-obtained dimethyl-2,5-thiophenedicarboxylate, in the form of white powder, was repeatedly washed with cold methanol and dried under vacuum before use.

High molecular weight thiophene-based polyesters such as PBTF, PPeTF, and PHTF were synthesized through two-step melt polycondensation, by treating dimethyl-2,5-thiophenedicarboxylate with different diols (in molar amount 1:3 with respect to the dimethyl ester) in the presence of titanium tetrabutoxide and titanium isoporopoxide catalysts (200 ppm each). Furthermore, all the reagents were charged in a 500-ml glass reactor, which was put in a silicon oil bath heated to 180°C. The reaction mixture was kept under nitrogen atmosphere and stirred at 100 rpm. When more than 90% of theoretical methanol was distilled off (about 2 h after diester dissolution), pressure was gradually reduced to 0.1 mbar, while the temperature was increased to 200°C. At the end of this second phase, which lasted two additional hours after maximum vacuum was applied, the temperature was set to 220°C for 15 min, afterward the reaction was stopped and the polymers were discharged.

The as-synthesized polymers were dissolved in chloroform and then purified by precipitation in cold methanol. The purified homopolymers were dried at room temperature for 48 h before further characterization and processing. Thin films (150 μm thick) were obtained by compression molding using a laboratory press (Carver), by melting the purified polymers between two Teflon sheets and applying a pressure of 5 tons/m^2^. The films were then ballistically cooled to room temperature in press.

### 2.2 Molecular, Thermal, and Structural Characterization

The chemical structure of polymers was checked by ^1^H-NMR spectroscopy at room temperature using a Varian Inova 400-MHz. Each sample was solved in deuterated chloroform with a concentration of 10 mg/ml.

TGA was carried out under inert atmosphere (N_2_ flow 30 ml/min) through a PerkinElmer TGA7, by heating about 5 mg of polymers at a rate of 10°C/min, in the temperature range between 40 and 800°C.

A PerkinElmer DSC6 was used for the calorimetric measurements. Samples weighing ~8 mg were heated at a rate of 20°C/min from −40°C to a temperature 40°C above the melting one (first scan), held for 3 min, then rapidly cooled (100 °C/min) to −40°C and held there for other 10 min. Finally, they were subjected to the same heating rump as the first scan (second scan).

As to X-ray diffraction (XRD) patterns, scans of films were collected in Bragg–Brentano geometry by means of a Malvern Panalytical MRD system equipped with a copper source (l = 0.15418 nm). For each step of 0.1° 2-theta was investigated for 25 s with a multi-channel solid state detector in the range 5.0°–60°.

### 2.3 Structure of Expression Vectors, Transformation, and Expression of Cutinases From *Thermobifida cellulosilytica* in *Escherichia coli*

The genes encoding Thc_cut1 and Thc_cut2 were cloned into the commercial expression vector pET-26b and hence fused at their 3’ with a sequence encoding for a 6xHis-Tag. The gene construct is preceded by the T7 promoter and is recognized by T7 RNA polymerase, whose gene is present in engineered strains of *Escherichia coli*, such as BL21, and whose synthesis is induced by isopropyl β-D-1-thiogalactopyranoside (IPTG). The two constructs were named pET-[cut1] and pET-[cut2] and used to transform *E. coli* BL21-Gold D3.

The preparation of each expression vector (10 μl, corresponding to ca. 20 ng) was added to a suspension of *E. coli* BL21-Gold D3 (100 µl) from a cell culture which had achieved the exponential phase and an optical density at 600 nm (OD600) of 0.3–0.5. Transformation was performed using the heat shock procedure (45 s at 42°C). Then, 900 µl of LB medium (10 g/L tryptone, 10 g/L NaCl, and 5 g/L yeast extract) were added and the cells were grown for 1 h at 37°C under agitation at 300 rpm. The whole cell suspension was then plated on two nutrient agar (Merck KGaA, Darmstadt, Germany, EMD Millipore Corporation) plates supplemented with kanamycin (40 μg/ml) as the antibiotic. A 100-µL aliquot was directly withdrawn and spread on a plate; the remaining cells were centrifuged at 10,000 rpm for 3 min and inoculated on the agarized medium after being resuspended in 100 µl of supernatant. Plates were incubated at 37°C overnight. Single colonies grown on the plate were picked and inoculated in 30 ml LB-kanamycin medium in 100-ml shaking flasks and incubated overnight at 37°C and 150 rpm on an orbital shaker to obtain a “preculture.” Afterward, 20 OD600 of preculture cells were transferred into 200 ml of LB-kanamycin medium in a 500-ml shaking flask, thus giving rise to an “expression culture.” The protein production was induced by adding 10 µl of IPTG, when the culture OD600 reached values between 0.6 and 0.8, and after the culture was cooled down to 20°C. The induced culture was incubated overnight at 20°C and 150 rpm. The cells were harvested by centrifugation (3,200 g, 4°C, 15 min), and the pellet was stored at −20°C until further use.

### 2.4 Enzyme Purification

The cell pellet was resuspended in binding buffer (20 mM sodium phosphate buffer pH 7.4 and 10 mM imidazole, 500 mM NaCl), in the measure of 5 ml buffer/g pellet and sonicated with four-times 45 s pulses, 60% amplitude under ice using a cell disruptor (BRANSON Ultrasonics). The cell crude extract was clarified by centrifugation (3,200 g, 4°C, 15 min), and the proteins of interest were purified from the soluble fraction. Proteins were purified by affinity chromatography on a ready-to-use 5 ml His-trap-FF column operated with the liquid chromatography system ÄKTAdesign™ (GE Healthcare). For this reason, the 6xHis peptide was fused over an Ala-Leu-Glu linker sequence to the C-terminal of the cutinases. The elution buffer was 20 mM sodium phosphate buffer pH 7.4, 500 mM imidazole, and 500 mM NaCl.

For the characterization and storage of cutinases, the elution buffer was exchanged with 100 mM Tris-HCl pH 7.0 with the use of PD-10 desalting columns (Amersham Biosciences) using the gravity protocol. In brief, the column was preconditioned with 25 ml 0.1 M Tris-HCl buffer pH 7.0 before 2.5 ml of protein samples were applied. Proteins were finally eluted in 3.5 ml of 0.1 M Tris-HCl pH 7.0.

Afterward, the purified protein solution was concentrated using Vivaspin 20 centrifugal concentrators 10,000 Da cut off (4,000 rpm for 30 min at 4°C). Enzyme aliquots were stored at −20 °C. The protein solution was aliquoted and stored at −20°C. Before their use, new assays of proteins and activities were carried out to verify that no denaturation event had occurred.

### 2.5 Protein Quantification With NanoPhotometer® and SDS-PAGE Analysis

Protein concentrations were determined using a NanoPhotometer® (Implen GmbH, Germany), based on molecular weight (Thc_cut1 = 29,441 g/mol and Thc_cut2 = 29,450 g/mol) and extinction coefficient (Thc_cut1 = 36,900 1/cmM and Thc_cut2 = 38,390 1/cmM) of the cutinases. SDS-PAGE was performed according to the Laemmli method using a precasted gel and loading 7 µl/well of each sample, along with 5 µl/well of protein marker (peqGOLD Protein Marker IV, VWR International, LLC). Gels were stained with Coomassie Brilliant Blue R-250. All measurements were carried out in triplicates.

### 2.6 Esterase Activity Assay

Spectrophotometric measurements of the activity were performed using para-nitrophenyl butyrate (p-NPB) as the substrate and followed by the development of para-nitrophenolate ion (absorption peak at 405–410 nm). The whole procedure consists of separately and sequentially preparing a “substrate solution”—also called “solution A”—and a “reaction solution”—also called “solution B”—to which the enzyme is added just before the reaction starts.

Furthermore, solution A consists of 86 µl of pNPB and 1,000 µl of 2-methyl-3-butanol; solution B consists of 40 µl of solution A and 1,000 µl of 0.1 M sodium phosphate pH 8. Finally, 20 µl of enzyme solution was added to 200 µl of solution B to start the reaction. The increase of the absorbance was measured at 405 nm, 30°C, for 5 min, every 18 s, using 96 well plates (Greiner 96 Flat Bottom Transparent Polystyrene) and a Tecan plate reader (Tecan, Grödig, Austria, Infinite M200 PRO).

### 2.7 Enzymatic Hydrolysis of TPCA-Based Polyesters

The polyesters were processed in the form of films of different thicknesses ranging from 102 to 236 μm. Pieces of 1 × 0.5 cm were obtained from each film. To remove possible impurities from the polymer surface, three washing steps were performed: the polymers were first incubated with Triton X-100 (5 g/L), Na_2_CO_3_ (100 mM), and ultrapure water. Each washing step was conducted at room temperature stirring at 50 rpm for 30 min. Then, each sample was dried overnight under a fume cupboard at room temperature. Afterward, the films were incubated horizontally in an orbital shaker with 1 ml of 1 M potassium phosphate buffer pH 8, with 5 µM Thc_cut1 or Thc_cut2. Incubations were performed at 50°C and 65°C, under orbital agitation of 150 rpm for 4 days. The samples were withdrawn after 24, 48, 72, and 96 h. All reactions were performed in quadruplicate. Blank reactions were performed in the same conditions, without adding the enzyme.

### 2.8 Weight Loss Determination

To evaluate the weight loss of the polymer film, each sample was weighed using a scientific balance with an accuracy of ±0.0001 g (Sartorius Lab Instruments, MSA225P-1CE-DI, Germany) before and after the hydrolysis reaction. The relative Eppendorf tube in which the reaction was carried out was also weighed for each sample.

$$Weight\;loss\left(\%\right)=100-\frac{Polymer\;final\left(mg\right)}{Polymer\;initial\left(mg\right)}\times 100.$$

### 2.9 High Performance Liquid Chromatography (HPLC-DAD)

After the enzymatic hydrolysis of the polyester film, the enzyme was removed following the ice-cold methanol precipitation protocol (volumetric ratio 1:1). The samples were centrifuged (Centrifuge 5427 R, Eppendorf AG, Hamburg, Germany) at 12,700 rpm at 4°C for 15 min. The supernatant was filtered through 0.2 µm PTFE filters and filled into HPLC vials. For HPLC (Agilent Technologies, 1260 Infinity, Palo Alto, CA, United States) measurements, a reversed phase column C18 (Poroshell 120 EC-C18 2.7 μm 3.0 × 150 mm) was used. Analyses were carried out using methanol (MeOH, phase A) and formic acid (HCOOH, phase B) gradient (see Supplementary Table S1 for details). The flow rate was set to 0.35 ml min^−1^ at a constant temperature of 40°C. The injection volume was 10 µl. The released products were measured with a photodiode array detector (Agilent Technologies, 1290 Infinity II, Vienna, Austria) at the wavelength of 260 nm. A preparation of 2,5-thiophenedicarboxylic acid was used to obtain different concentrations (from 0.001 to 1 mM) and loaded as previously described for HPLC samples to obtain a calibration curve and to quantify the eluted dicarboxylic acids. A post time of 4 min was used to equilibrate the column.

### 2.10 Fourier Transform Infrared Spectroscopy

The polymer film surfaces before and after hydrolysis were characterized using a PerkinElmer Spectrum 100 FT-IR Spectrometer. Spectra were collected at a resolution of 4 cm^−1^ for 32 scans. All spectra were recorded at room temperature over the wavelength interval between 4,000 and 650 cm^−1^. The normalization was made on the peak at 1703 cm^−1^ that corresponds to the C=O bond of the carbonyl group of the carboxylic acid and it is not changing with the hydrolysis catalyzed by the cutinases. The bands were assigned as follows: 1,236 cm^−1^ ν (C-O-C), 1,081 cm^−1^, and 1,030 cm^−1^ ν (C-O). Normalization was based on the peak at 1,073 cm^−1^ assigned to ν (C=O).

### 2.11 Liquid Chromatography-Time-of-Flight/Mass Spectrometry

Liquid chromatography-time-of-flight/mass spectrometry (LC-TOF/MS), in positive ionization mode, was used to qualitatively identify the released soluble oligomers. The analytes were separated using an HPLC (1260 series, Agilent Technologies, Palo Alto, CA) equipped with a reversed-phase C18 rapid resolution column (Zorbax Eclipse XDB, Agilent Technologies) of 50 mm by 2.1 mm and 1.8 µm particle diameter. Column temperature was 40°C. Mobile phase A consists of 20 mM ammonium formiate NH_4_COOH in ultrapure H_2_O and mobile phase B was ultrapure acetonitrile (see Supplementary Table S2 for details).

The flow rate was 0.5 ml min^−1^ and the injection volume was 20 µl. This HPLC system was connected to a time-of-flight mass spectrometer (6230 TOF LC/MS, Agilent Technologies) equipped with an electrospray interface under the following operating parameters: capillary 3500 V, nebulizer 40 psig, drying gas 8 L min^−1^, gas temperature 300°C, fragmentator 125 V, skimmer 65 V, and OCT 1 RF Vpp 750 V. The mass axis was calibrated using the mixture provided by the manufacturer over the m/z 50–3,200 range. A second orthogonal sprayer with a reference solution was used as a continuous calibration using the following reference masses: 121.050873 and 922.009798 m/z. Spectra were acquired over the 50–3,000 m/z range at a scan rate of two spectra per second.

### 2.12 Scanning Electron Microscopy

The morphology of polyester films was qualitatively assessed through scanning electron microscopy (SEM). Control polyester films (without any enzymatic treatment) and enzymatically hydrolyzed films (after 24, 48, 72, and 96 h) were surface characterized. All SEM images were acquired by collecting secondary electrons on a Hitachi 3030TM (Metrohm INULA GmbH, Austria) working at EDX acceleration voltage.

### 2.13 Gel Permeation Chromatography

Samples were dissolved in CHCl3 at a concentration of 2 mg ml^−1^ and filtered through cotton prior to addition in a HPLC vial. The PBTF samples were instead dissolved in a mixture of 10% hexafluoro isopropanol in CHCl_3_. Gel permeation chromatography was carried out at 30°C on an Agilent Technologies HPLC System (Agilent Technologies 1260 Infinity) connected to a 17,369 6.0 mm ID × 40 mm L HHR-H, 5 μm Guard column and a 18,055 7.8 mm ID × 300 mm L GMHHR-N, 5 μm TSKgel liquid chromatography column (Tosoh Bioscience, Tessenderlo, Belgium) using 1 ml min^−1^ CHCl_3_ as mobile phase. An Agilent Technologies G1362A refractive index detector was employed for detection. The molecular weights of the polymers were calculated using linear polystyrene calibration standards (Polystyrene standard ReadyCal set Mp 400–2,000,000, Sigma-Aldrich).

## 3 Results and Discussion

### 3.1 Polymer Synthesis and Characterization

**FIGURE 1:**
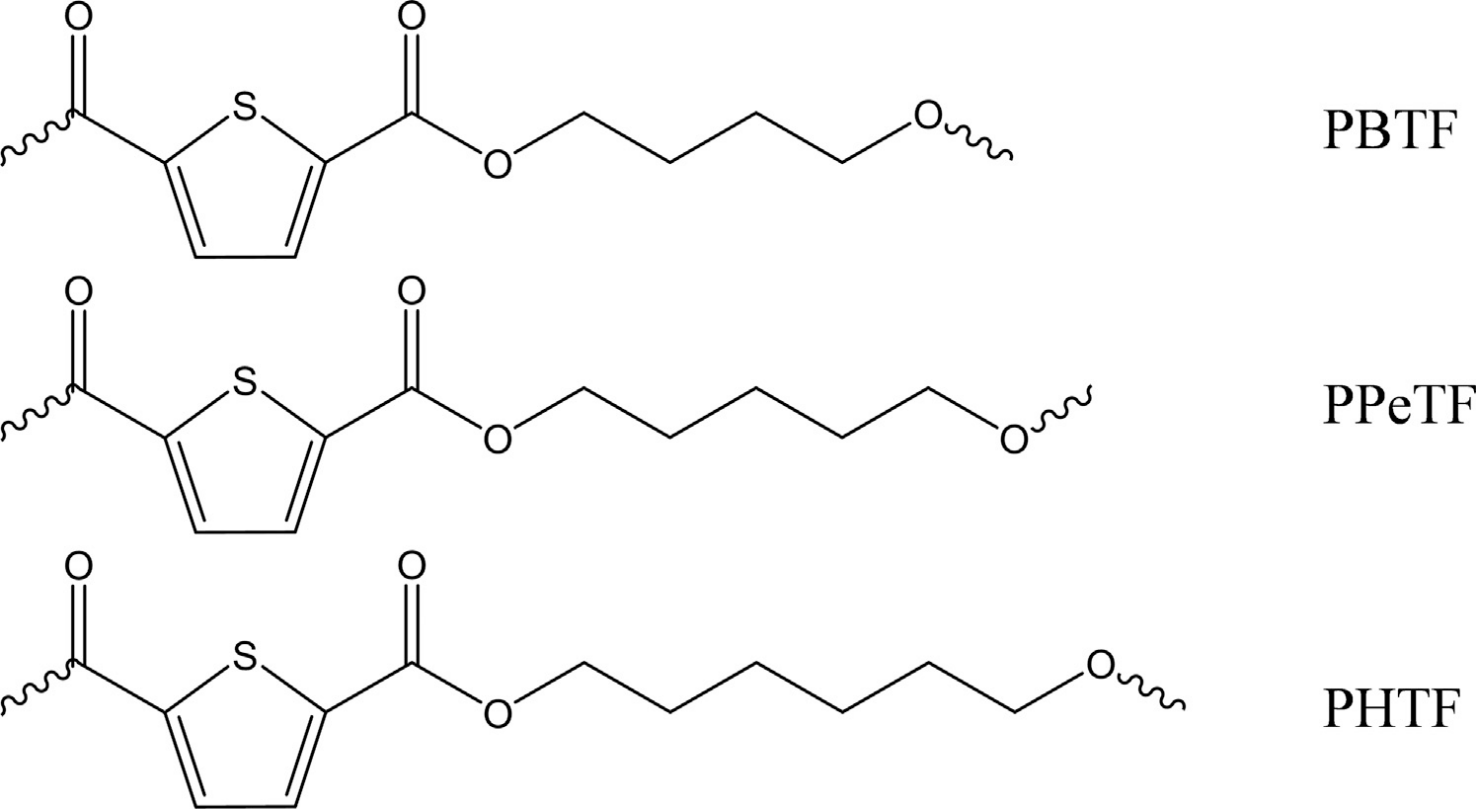
Chemical structure of PBTF, PPeTF, and PHTF.

Poly(butylene 2,5-thiophenedicarboxylate) (PBTF), poly(pentamethylene 2,5-thiophene dicarboxylate) (PPeTF), and poly(hexamethylene 2,5-thiophenedicarboxylate) (PHTF) polymers appeared like light brown-colored solids, while after purification they formed white floccules. Their chemical structure (Figure 1) was confirmed by ^1^H-NMR analysis (Figure 1). In the case of PBTF, the hydrogen atoms of 1,4-butane moiety (4H, m) and (4H, t) can be found at δ 2.00 ppm and δ 4.48 ppm, respectively. As to the PPeTF spectrum, the methylene protons of the aliphatic subunit (2H, m), (4H, m), and (4H, t) were located at δ 1.58 ppm, δ 1.82 ppm and δ 4.34 ppm, respectively, while in the case of PHTF spectrum, the hydrogen atoms of glycolic subunit (4H, m), (4H, m), and (4H, t) can be detected at δ 1.51 ppm, δ 1.81 ppm, and δ 4.32 ppm, respectively. In all cases, the singlet of thiophene ring was located at δ 7.70 (PPeTF and PHTF) or 7.83 ppm (PBTF). No additional peaks were found, proving no impurities were present in the samples.

**FIGURE 2:**
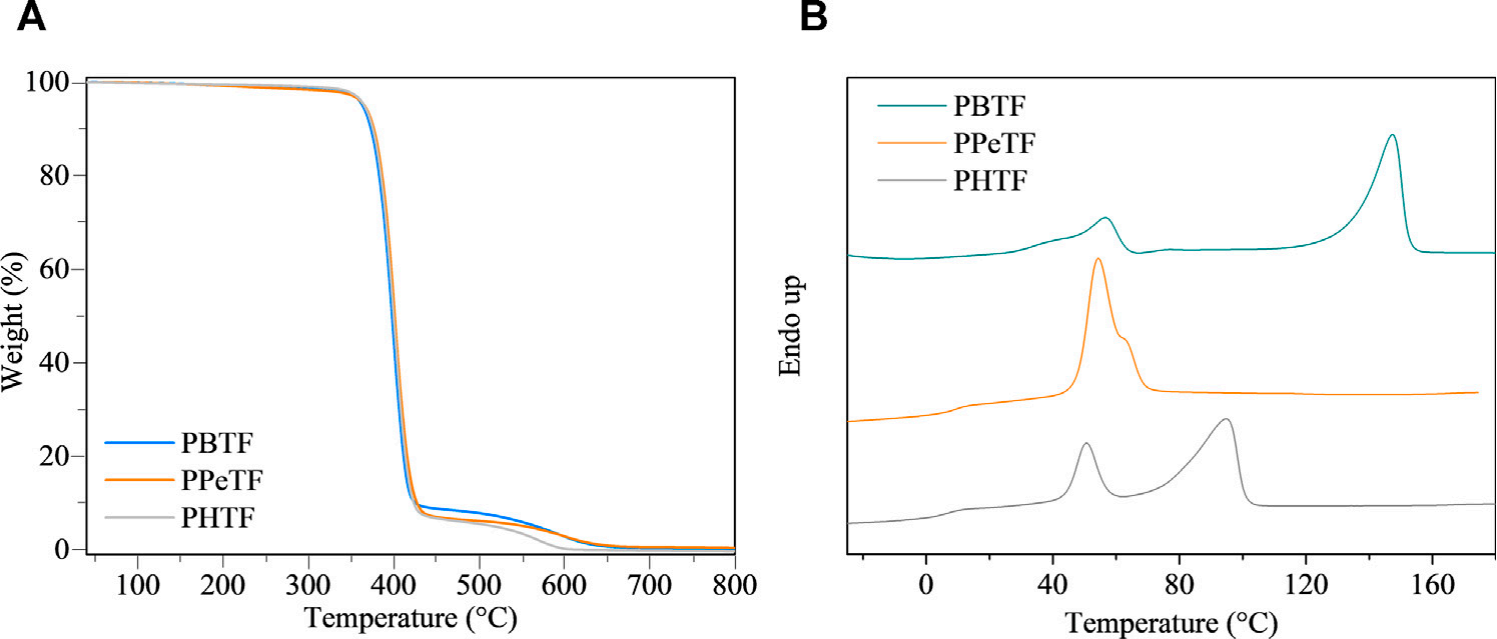
**(A)** Thermogravimetric curves under nitrogen atmosphere (gas flow 30 ml/min and heating rate 10°C/min) and **(B)** first DSC scan curves of the homopolymers under investigation.

The polymers were compression molded to form films of thickness around 150 μm. The results of thermogravimetric analysis (TGA) are reported in Table 1 and shown in Figure 2A. It can be observed that all the materials are characterized by high and comparable thermal stability, T_onset_ ranging between 376 and 379°C and T_max_ varying from 400 to 402°C. In all cases, degradation occurs in two steps, suggesting the same decomposition process. No residual char was present at 800°C.

**TABLE 1:** Thermal characterization data (TGA and DSC) of the thiophene-based polymers described in this work.

	TGA		First DSC scan						Second DSC scan					
	T_onset_ °C	T_max_ °C	T_g_ °C	∆c_p_ J/g°C	T_1_ °C	T_2_ °C	∆H_1_ J/g	∆H_2_ J/g	T_g_ °C	∆c_p_ J/g°C	T_c_ °C	∆H_c_ J/g	T_m_ °C	∆H_m_ J/g
PBTF	376	400	n.d	n.d	55	147	5	29	25	0.237	88	28	147	28
PPeTF	380	402	8	0.123	56	65	24	6	8	0.308	—	—	—	—
PHTF	379	402	6	0.132	51	95	9	25	1	0.359	—	—	—	—

First DSC scan profiles are shown in Figure 2B, the corresponding results being listed in Table 1. As it can be seen, DSC traces show a baseline deviation due to glass-to-rubber transition followed by endothermic phenomena at higher temperature. In the case of PBTF, the low intensity endothermic phenomenon occurring at lower temperature around 55°C (associated heat around 4 J/g), just above glass transition phenomenon, has been previously associated to a mesophase (a 1-D/2-D order phase) arising from π–π stacking among thiophene rings (Guidotti et al., 2018b; Guidotti et al., 2018c). The most intense endothermic process at higher temperature is instead due to the melting of the crystalline portion of the film (Guidotti et al., 2018b; Guidotti et al., 2018c). The endothermic phenomenon present in the PPeTF DSC trace is broad (the process starts at around 45°C) and asymmetric, suggesting it results from the superimposition of two endothermic phenomena. The most intense and lowest temperature peak could be presumably associated with a meso form coexisting with crystals characterized by a low degree of perfection (melting at 56°C), the highest temperature (≈65°C) shoulder on the contrary due to the melting of the crystalline portion of the material. The heats associated to the two phenomena are reported in Table 1. Lastly, in the case of PHTF, two well-separated endothermic phenomena are present: one at lower temperature starting at around 40°C and centered at 51°C, again, due to meso phase and poor crystallinity, the other at around 95°C ascribable to the melting of crystalline phase.

From the comparison of melting temperature values, the polymer containing an odd number of methylene groups in the glycol subunit presents the lowest Tm; considering the two samples with even number of carbon atoms in the aliphatic segment, it can be noted that the Tm value decreased with glycol subunit length. As far as the expected Tm trend is concerned, polymers containing longer aliphatic chains (glycol subunit) are more flexible, thus crystallizing more readily and melting at higher temperature. The observed trend on the contrary, evidenced an even-odd effect, the polyesters with an even number of carbon atoms per repeat units, i.e., PBTF and PHTF with 4 and 6 per repeat units, respectively, having higher melting points than PPTeF, which is the only one characterized by an odd number of carbon atoms (de Jong et al., 2012).

Therefore, flexibility is not the only parameter that has to be taken into consideration, but also chain symmetry and conformation (Guidotti et al., 2018d).

**FIGURE 3:**
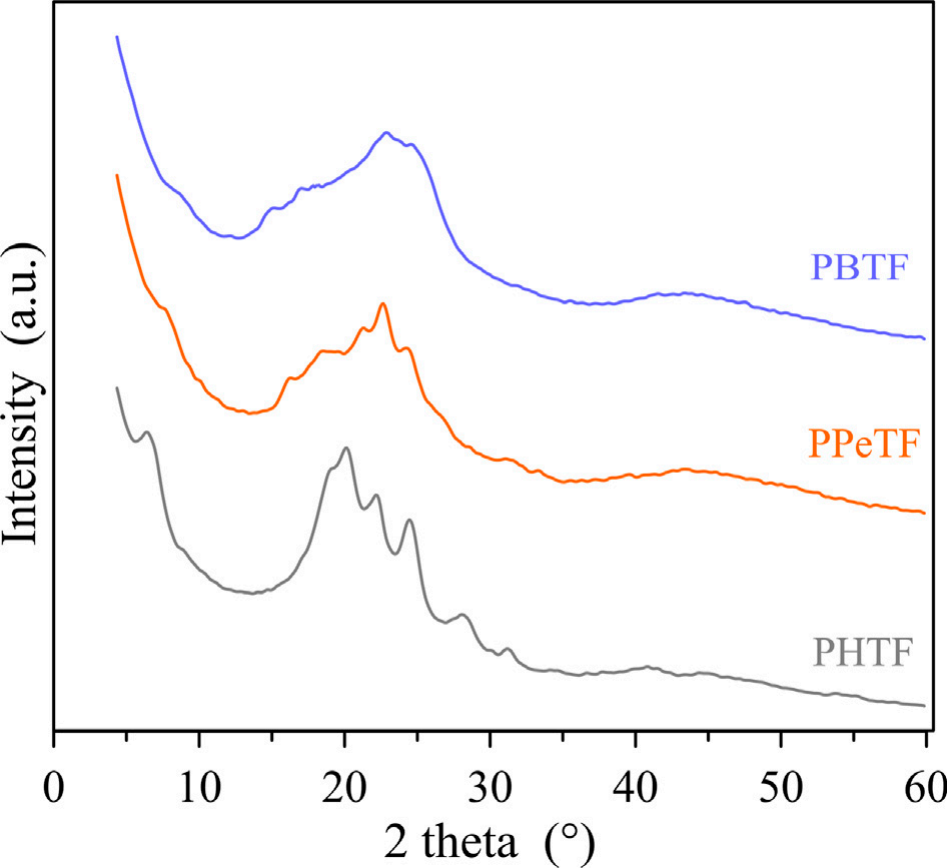
XRD patterns of the investigated polyesters; interlayer distances of the main peaks are indicated in Angstrom.

As for the crystallinity degree, as expected the even C number containing glycol subunit are the most crystalline, PBTF film being slightly more crystalline than PHTF. In the second DSC scan (Table 1), after rapid cooling from the molten state, in all cases the materials turned out to be amorphous, although with some differences. In fact, PBTF behavior is typical of an amorphous material able to crystallize during heating, while for PPeTF and PHTF only baseline deviation due to glass-to-rubber transition is present. As far as the glass transition value is concerned, it regularly decreased with glycol sub-unit length, as expected, considering the well-known flexibilizing effect imparted to the polymer chain by methylene group segments. From the Tg values measured in the second scan it is evident that at room temperature the amorphous phase of PBTF film is not completely mobile, whereas PPeTF and PHTF films are in the rubbery state. If we consider the enzymatic experiments were carried out at 50 and 65°C, all the samples investigated are in the rubbery state, thus they can evolve with time. To gain more insight into sample microstructures, film samples have been subjected to X-ray diffraction (XRD). At first sight, the patterns (Figure 3) appeared quite different. PBTF film shows only broad peaks of low intensity overlapped to a bell-shaped intense background. Relative maxima can be seen at 15.1°, 17.0°, 22.9°, and 24.7° and are compatible with crystalline α-PBTF (Guidotti et al., 2018c).

PPeTF sample shows broad peaks or shoulders at 7.5°, 16.3°, 18.4°, 21.3°, 22.6°, and 24.3°C. The PHTF profile is characterized by well defined peaks identified at 6.5°, 19.2°, 20.0°, 22.2°, 24.4°, 28.1°, and 31.3 C°, clearly indicating an elevated crystallinity of the sample. This pattern is comparable with the one reported by other authors (Guidotti et al., 2018c).

### 3.2 Enzymes Expression, Purification, and Characterization

The enzymes *Thermobifida cellulosilytica* cutinase 1 (Thc_cut1) and 2 (Thc_cut2) were produced as recombinant proteins using *E. coli* as the host. For both proteins, the expression was maximum after 20–24 h from induction (Supplementary Figure S1). The purification of the His-tagged Thc_cut1 and Thc_cut2 was carried out on the soluble fraction via IMAC using a chromatograph and analyzed by SDS-PAGE indicated a >95% purity (Supplementary Figure S2). The production and purification yield from 200 ml in-flask culture were 26.98 ± 3.74 and 33.79 ± 4.84 mg for Thc_cut1 and Thc_cut2, respectively (Supplementary Figure S3). As a total, 433 mg of purified Thc_cut1 and 653 mg of purified Thc_cut2 were produced.

The activity of Thc_cut1 and Thc_cut2 was assessed based on the hydrolysis of a small model molecule containing an ester bond, namely, para-nitrophenyl butyrate. The two enzymes showed a comparable activity, with 141.66 ± 32.18 U/mg and 159.91 ± 55.84 U/mg activity for Thc_cut1 and Thc_cut2, respectively (Supplementary Figure S4).

### 3.3 Polyester Hydrolysis

The 2,5-thiophenedicarboxylic acid-based polymers, PBTF, PPeTF, and PHTF, were subjected to enzymatic hydrolysis by the recombinant Thc_cut1 and Thc_cut2.

**FIGURE 4:**
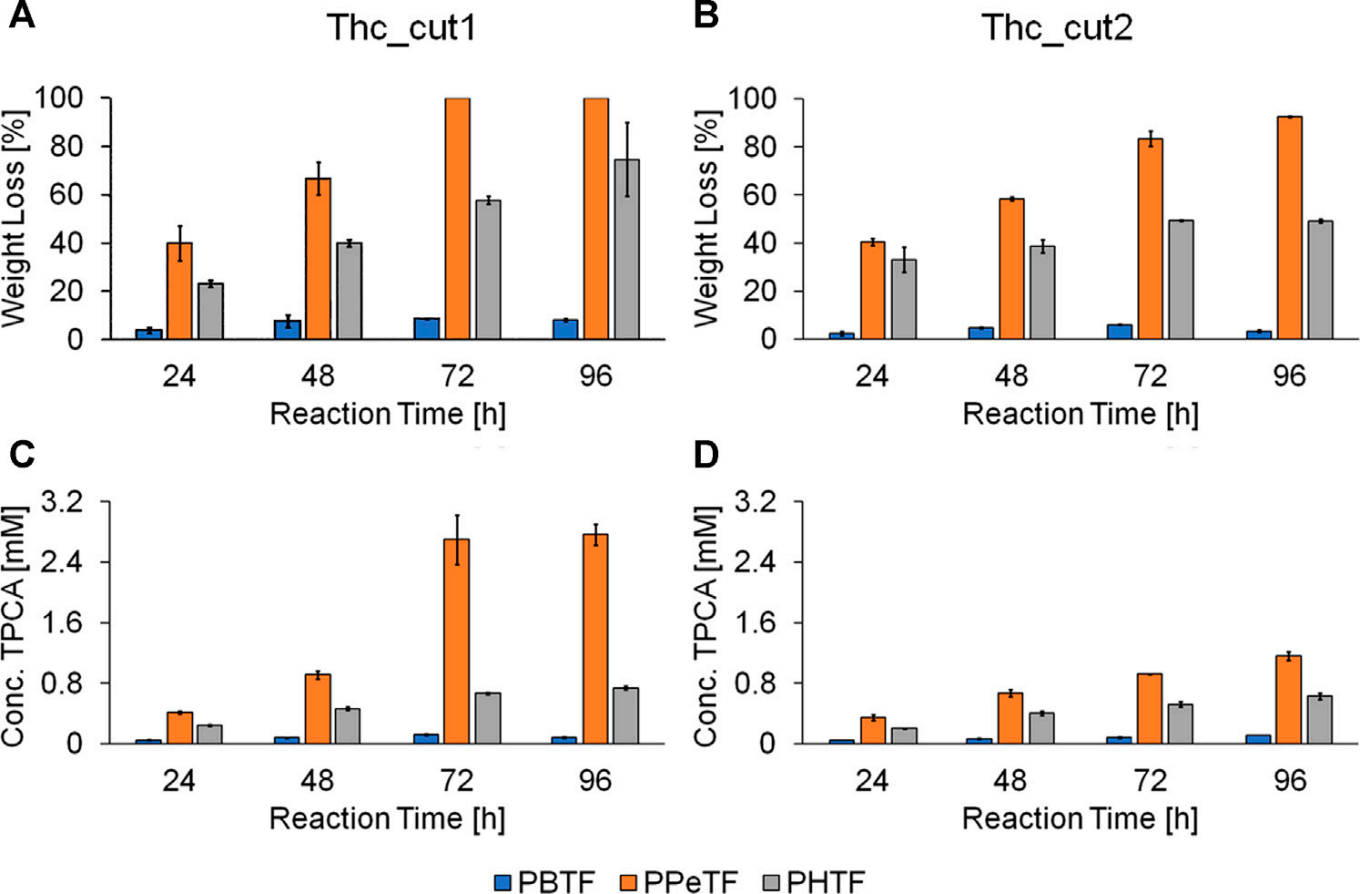
Enzymatic hydrolysis of TPCA-based polyesters. Weight loss (panels **A** and **B**) and HPLC analysis (panels **C** and **D**) of the polyester films treated with Thc_cut1 (panels **A**, **C**) and Thc_cut2 (panels **B** and **D**) using 1 M KH_2_PO_4_/K_2_HPO_4_ buffer pH 8.0 at 65°C. All experiments were performed in quadruplicate and the average values ± the standard deviations are shown.

Both enzymes were more active on the TPCA-based polymers at 65°C than at 50°C (Figure 4 and Supplementary Figures S5–S8). This observation is consistent with previously reported data for the hydrolysis of poly(ethylene 2,5-furanoate) (PEF) amorphous films (55% weight loss in 96 h) (Gamerith et al., 2017) and PET. Compared to PET, PPeTF was more efficiently degraded: indeed, we observed a 100% weight loss with Thc_cut1 and a weight loss >80% withThc_cut2 after incubation for 72 h at 65°C (Figures 4A,B, orange bars). Notable results were also obtained with PHTF, polyester containing the 6-carbon diol. In this case, a weight loss of ∼80 and ∼50% after 96 h at 65°C was measured with Thc_cut1 and Thc_cut2, respectively (Figures 4A,B, grey bars). The degradability of PBTF was also assessed, but the measured weight losses (Figures 4A,B, blue bars) were considerably lower (50%).

The observed trend (PPeTF degrades faster than PHTF, which is degraded faster than PBTF) can be explained considering several factors affecting the kinetics of the degradation process, such as molecular weight, ester group density, hydrophilicity, crystallinity degree, amorphous phase mobility, and melting temperature (Gigli et al., 2019).

Specifically, the higher the ester group density, the shorter the glycol subunit, the hydrophilicity, and the amorphous phase mobility, i.e., the lower the Tg, and the lower the crystallinity degree and the melting temperature, the faster the degradation rate. Comparing the three polyesters under investigation, the ester group density, the hydrophilicity, and the glass transition temperature decrease in the order PBTF>PPeTF>PHTF, while the melting temperature follows an even/odd trend with PPeTF sample showing the lowest melting temperature. Lastly, the crystallinity degree changes according to the following trend: PBTF>PHTF>PPeTF.

However, it has to be taken into consideration that the degradation experiments have been carried out at higher temperatures (50 and 65°C) than room temperature. At those temperatures, all the investigated samples are characterized by a mobile amorphous phase, the different behaviour in terms of degradability being therefore mainly determined by the crystal phase characteristics. PPeTF resulted to be the fastest degraded polymer (being the least crystalline and having the lowest melting temperature).

The slowest degrading polyester is PBTF mainly because of its high melting crystalline phase, the crystallinity fraction being only slightly higher than that of PHTF.

Furthermore, PBTF was degraded to a significantly lower extent than previously reported for Thc_cut1 after 72 h at 65°C (Guidotti et al., 2018a). The difference can be ascribed to the semicrystalline nature of the PBTF film investigated in the present paper. The film previously studied was on the contrary completely amorphous (Guidotti et al., 2018a). As mentioned above, crystallinity makes the enzyme attack more difficult, due to the lower accessibility of polymer chains (Guidotti et al., 2018a).

After the gravimetric determination, gel permeation chromatography of both the neat and degraded film fragments was carried out to detect the size of polymeric chains of PBTF, PPeTF, and PHTF. Overall data indicate that there are no significant differences in chain length between samples of a given polymer before and after the enzymatic treatment (Supplementary Figure S9). These results are in line with recently published data on the hydrolytic degradation of poly(caprolactone) using *Candida antarctica* lipase (Soccio et al., 2007). The common interpretation is that the hydrolysis of the polymer occurs uniformly on its surface, therefore through a layer-by-layer mechanism, which also confirms the endo-wise activity on the accessible polymer chains previously observed by our group (Guidotti et al., 2018a). The overall effects could be also dependent on the specific polymer chain packing characteristics of each polymer type, which in turn depends on intrinsic/chemical features of monomers. For example, the higher aromaticity (i.e higher resonance energy) and lower dipole moment of thiophene ring compared to the furan one results in weaker and different polymer chain interactions in PBTF when compared to PBF. In the latter, stronger inter-chain hydrogen bonds are established (Guidotti et al., 2018a; Guidotti et al., 2018c; Wang et al., 2019).

Moreover, when comparing the alkaline and enzymatic hydrolysis of poly(lactic acid), it emerged that the alkali-based hydrolysis of the films led to a strong reduction of the polymer molecular weight (30% molecular weight decrement after just a 10 min treatment with NaOH). On the other hand, the enzymatic hydrolysis with a cutinase from *Humicola insolens* caused a minimal decrease of molecular weight (>5% after a 72 h treatment) (Bikiaris et al., 2008), which is consistent with our results.

### 3.4 Analysis of the Soluble Released Products

Besides monitoring the weight loss (Figures 4A,B), the film degradability of TPCA-derived polymers was followed analyzing the release of their degradation products (monomers and soluble oligomers) in the solution. Results indicate a higher concentration of released TPCA in samples incubated at 65°C rather than at 50°C for all polymers, in line with the trend observed for the weight loss analysis. The HPLC results also confirm the highest degradability of PPeTF compared to the other polymers. In fact, the reactions performed at 65°C using Thc_cut1 (72 h reaction time) led to a 2.70 mM concentration of released TPCA for PPeTF, a value that is about 4 times higher compared to the one measured for PHTF (0.67 mM) and even 22 times greater than what was obtained with PBTF (0.12 mM). The higher hydrolytic activity of Thc_cut1 vs Thc_cut2 is confirmed, comparing the concentrations of TPCA released with the two tested enzymes. For example, the hydrolysis of PPeTF performed at 65°C and 72 h led to the release of 2.70 and 0.92 mM of TPCA with Thc_cut1 and Thc_cut2, respectively. As expected, coherently with weight loss measurements, the HPLC results show a lower PBTF degradability compared to PPeTF and PHTF and also respected to the sample previously reported (Guidotti et al., 2018a). Again, this last result can be correlated to the semicrystalline nature of the PBTF film under investigation.

After quantifying the released TPCA, the enzymatic degradation of PPeTF and PHTF was also followed through the release of soluble oligomers using LC-TOF/MS analysis. The types of oligomers found in solution were AB, ABA, and BAB, where A indicates TPCA, and B the diol (C5 or C6 based on the discussed polymer). When analyzing the results, it must be considered that the oligomers are intermediates, which can be further hydrolyzed and transformed into TPCA monomer, thus increasing -or decreasing-in quantity over time due to the progression of the reaction. Overall, the presence of oligomers together with the release of TPCA confirms the enzyme has both an endo-wise and exo-wise hydrolytic activity.

**FIGURE 5:**
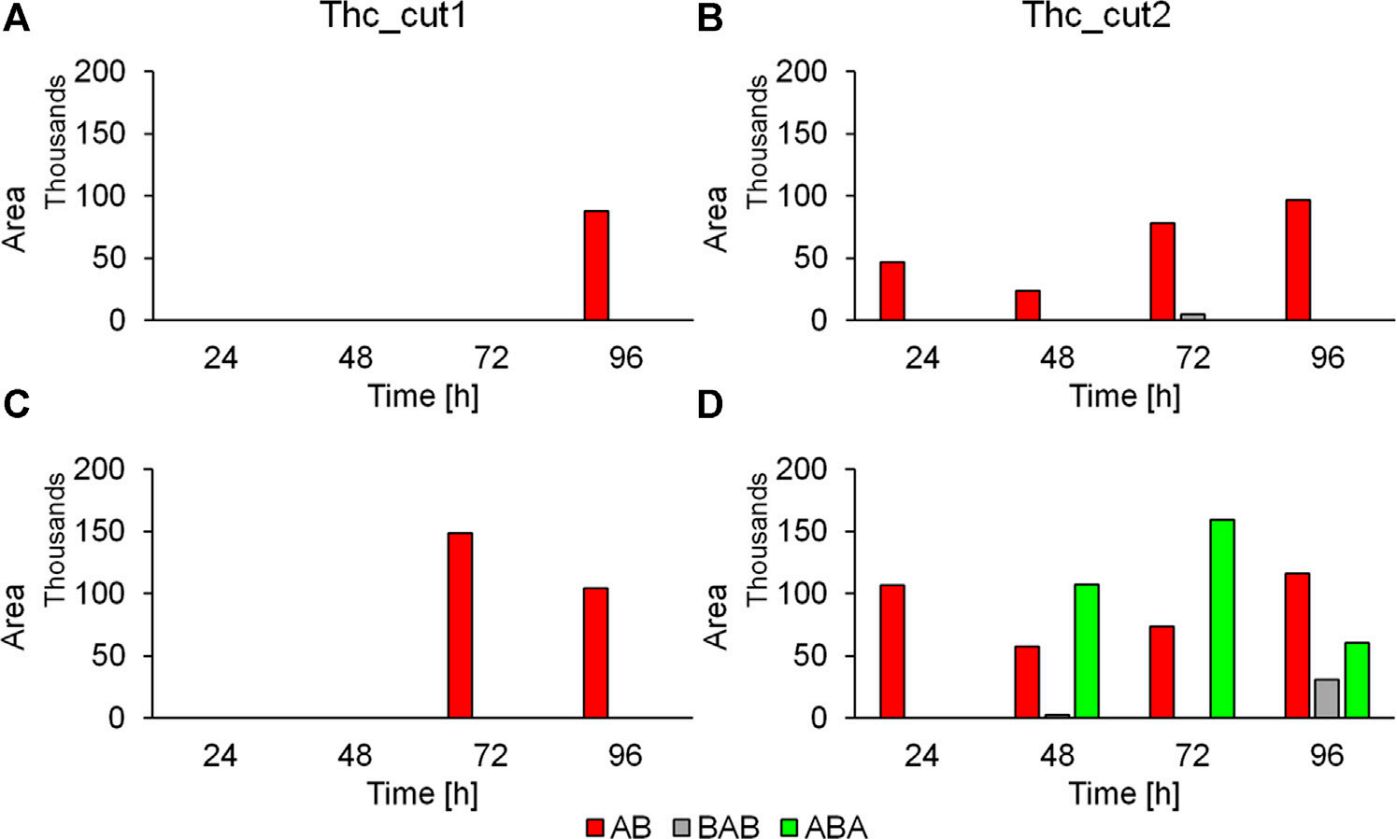
Released oligomers from the PPeTF hydrolysis determined via LC-TOF/MS analysis. Time course reaction showing the released dimers AB, ABA, and BAB for the enzymatic hydrolysis performed at 50°C (panels **A** and **B**) and 65°C (panels **C** and **D**) when using Thc_cut1 (panels **A** and **C**) and Thc_cut2 (panels **B** and **D**). The reactions were performed in 1 M KH_2_PO_4_/K_2_HPO_4_ buffer pH 8.0. No released products were detected in the control reactions.

In general, we can observe that Thc_cut1 produces smaller (AB) and less heterogeneous oligomers (ABA and BAB) than those generated by Thc_cut2 for both tested polymers (Figure 5). This can be attributed to the slower hydrolytic reaction of this enzyme. The presence of oligomers from PHTF treated at 50°C with Thc_cut2 (Supplementary Figure S10B), and not with Thc_cut1 (Supplementary Figure S10A), is another evidence highlighting the different rate of hydrolysis between the two enzymes. In case the reaction is carried out at higher temperature (65°C), the oligomers are most likely already hydrolyzed by Thc_cut1, as supported by quantitative HPLC analysis, showing a constant TPCA increase from 0.41 mM (PPeTF) and 0.24 mM (PHTF) after 24 h to 2.77 mM (PPeTF) and 0.74 mM (PHTF) after 96 h (Figure 4C).

The LC-TOF/MS data indicate that oligomers are present at higher concentration in the reaction mixtures when hydrolytic degradation is carried out at 65°C than at 50°C, most probably because Thc_cut1 is more active at 65°C. For instance, the AB dimer derived from PPeTF hydrolysis is strongly present (Area = 148870) after a 72 h reaction at 65°C (Figure 5C), but it was not detected at the same time point when the degradation was performed at 50°C using Thc_cut1 as catalyst (Figure 5A). Some oligomers, in particular the dimer AB, showed a rather fluctuating concentration trend as the reaction proceeds. As aforementioned, this could be due to the subsequent hydrolysis of oligomers into TPCA. This result is evidenced considering the concentration peak of AB oligomers derived from PPeTF after 72 h of reaction (Figure 5C): their concentration decreases with time and concomitantly an increase of the TPCA concentration is observed with HPLC (Figure 4C).

### 3.5 Polymers Surface Analysis

**FIGURE 6:**
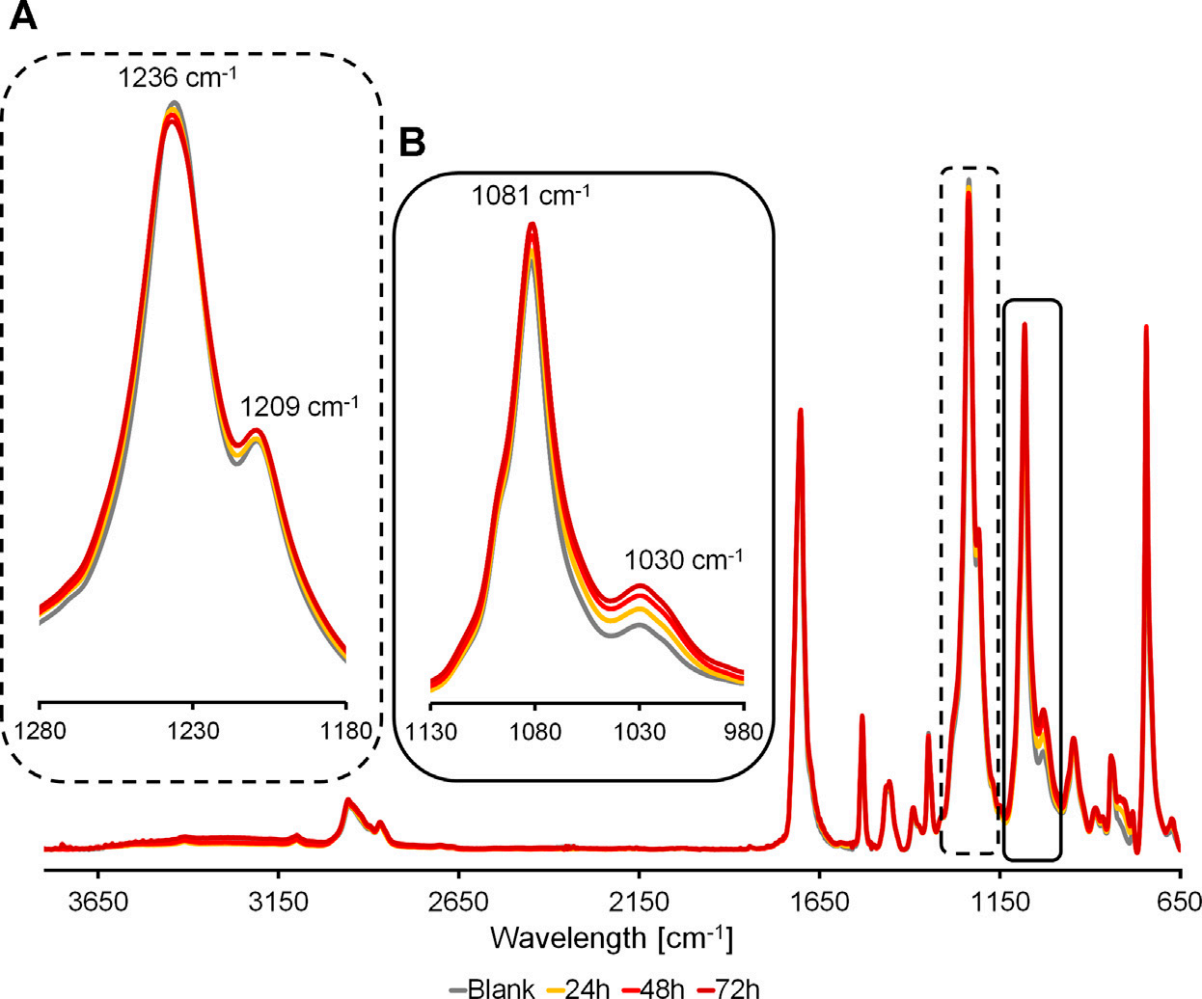
FT-IR spectra of partially hydrolyzed PPeTF films. Samples were treated with Thc_cut1 at 65°C for 48 h. Spectra have been normalized with respect to the intensity of the peak at 1703 cm^−1^. A: magnification of peaks correlated to the stretching of the C-O bond of a primary alcohol and B: magnification of peaks correlated to the stretching of the ester bond.

The partially hydrolyzed surfaces of the polymers were studied via FT-IR to highlight the effect of enzymatic activity on the polymer film surface. The FT-IR spectra acquired from the C4- and C6-polymers treated with cutinases (Supplementary Figures S11, S12) do not show significant differences with respect to the control reactions. This can be due to a marginal impact exerted on these materials by the two enzymes and due to their slow reaction kinetics, as demonstrated by both the weight loss and HPLC results. Instead, the FT-IR analysis of PPeTF, the polymer with the highest degradability according to the weight loss and HPLC analyses, highlights a marked increase of the 1,081 cm^−1^ and 1,030 cm^−1^ peaks as the reaction progresses (Figure 6B), while the peak at 1,236 cm^−1^ decreases (Figure 6A). The peaks at 1,081 cm^−1^ and 1,030 cm^−1^ are both correlated to the stretching of the C-O bond of a primary alcohol. This C-OH group can be formed by breaking of the ester bond and we can observe the corresponding FT-IR peak increases with the progression of the hydrolysis reaction over time. The signal at 1,236 cm^−1^ associated to the stretching of the ester bond hydrolyzed by the enzyme, decreases with time in agreement with the reduction of the ester bonds content in the material as the hydrolysis reaction proceeds.

**FIGURE 7:**
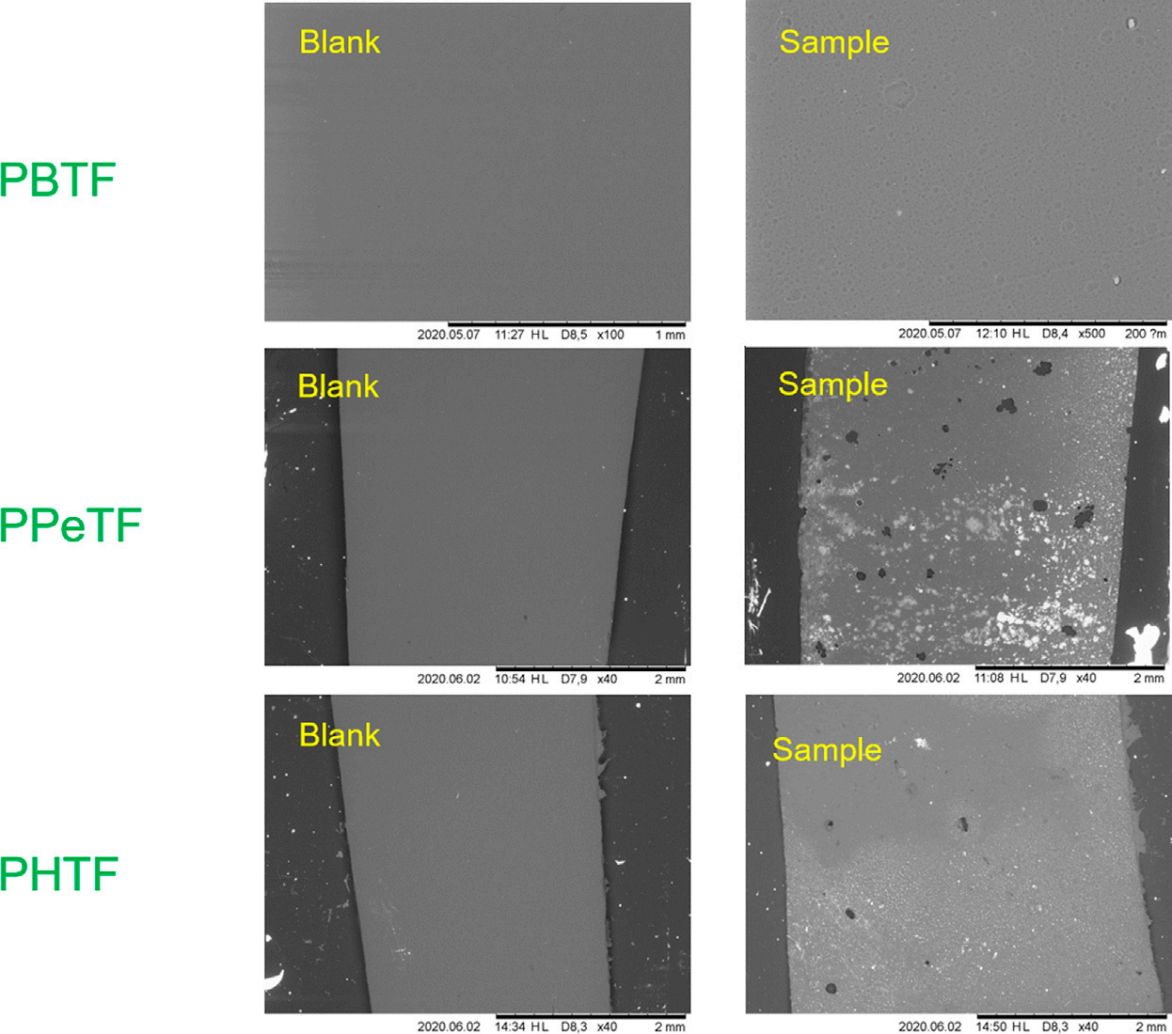
Scanning electron microscopy (SEM) imaging of TPCA-based polyester films after enzymatic hydrolysis. Right side shows the films after enzymatic hydrolysis with Thc_cut1 in 1 M KPO buffer pH 8 at 65°C; left side shows the control reactions.

SEM analysis of the polymer samples before the treatment and after 48 h of reaction using the two cutinases showed tiny holes on the film surfaces and confirmed that most effect is obtained for both enzymes at 65°C on PPeTF (Figure 7).

**FIGURE 8:**
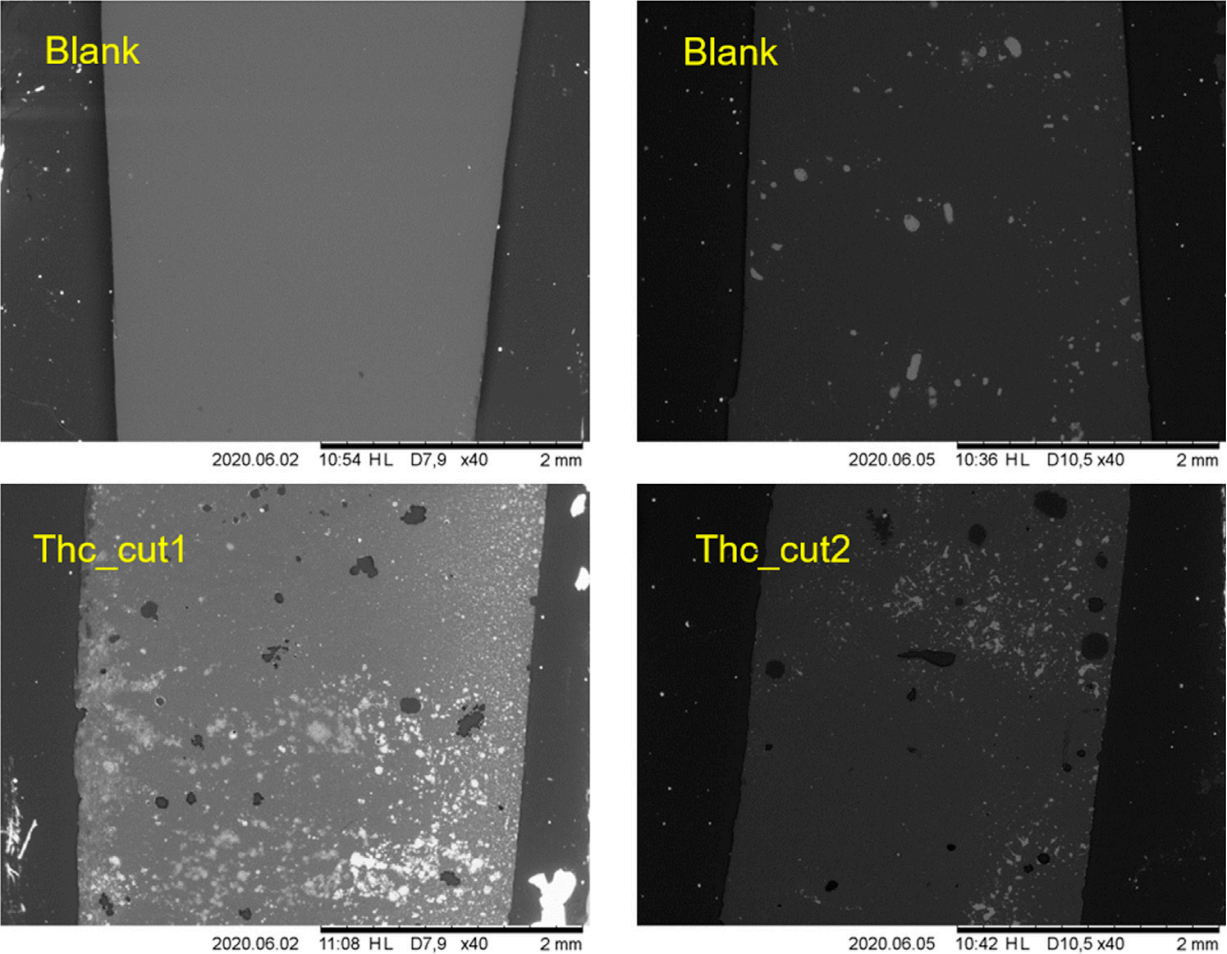
Scanning electron microscopy (SEM) imaging of PPeTF films after enzymatic hydrolysis in 1 M KPO buffer pH 8.0 at 65°C. Left side shows the films hydrolyzed with Thc_cut1; right side shows the films hydrolyzed with Thc_cut2. The upper pictures show the control reactions. Magnification: 40x.

In particular, Figure 8 shows the comparison between the effects exerted by Thc_cut1 and Thc_cut2 on PPeTF at 65°C after 72 h of reaction. From the images, we can evict both enzymes are active, since both cause the formation of visible holes on the polymer surface; however, with Thc_cut1 being more active than Thc_cut2, fully confirming the trends observed with the other analytic techniques aforementioned.

### 3.6 DSC and WAXS Analysis of Partially Degraded Samples

Supplemenatry Figure S13 and Supplementary Table S3 show the changes in crystallinity of PPeTF and PHTF polyesters before and after the degradation by cutinases at two different temperatures (PBTF was not considered due to its negligible weight loss). In Supplementary Figure S13, first DSC scan curves of partially degraded samples at different timepoints are reported, together with those of the corresponding blanks, while in Supplementary Table S3, melting temperature and the relative ΔH_m_ of partially degraded samples and blanks are listed.

The DSC curves of the partially degraded PPeTF and PHTF at different times during enzymatic degradation at 50 and 65°C do not show relevant variations, since the samples and the relative blanks are characterized by the same DSC profile, the slight deviation in T_m_ and ΔH_m_ values being within the experimental errors. Anyway, after only 24 h from the start of enzymatic degradation both the DSC profile of the partially degraded samples and of the corresponding blanks change significantly, respect to that of the compression molded film measured at room temperature. The permanence at 50 or 65°C for 24 h determines the disappearance of the peak at 55°C and the intensification of the peak at 65°C relative to the melting of crystalline phase, which becomes narrow and symmetrical to indicate a population of crystals with a more uniform degree of perfection. The only difference between the profiles at 50°C and those at 65°C is the position of melting peak, which is located at higher temperature when the enzymatic degradation experiments are carried out at 65°C. As known, both mesophase and poor crystalline phases, if suitably annealed, can evolve into a more perfect, thus higher melting, crystal phase.

In the case of PHTF, the profiles of partially degraded samples together with those of the corresponding blanks are more similar to the profiles of the undegraded compression molded film stored at room temperature. In fact, two endothermic phenomena are still evident, even though they appear to be less separated, due to the shift to higher temperatures of the endo peak at lower temperature.

The intensity ratio between the two endothermic peaks changes at both enzymatic degradation temperatures, the peak at lower temperature becoming even more intense at 65°C. Again, such results can be explained as due to conversion of the mesophase and less perfect crystals into more perfect 3-D ordered phase.

The increment of sample crystallinity degree regards in equal measure both blanks and enzymatically degraded samples indicating that the result is due to the permanence of films at a temperature above T_g_, where amorphous macromolecular chains are mobile and mesophase and poor crystals can evolve to more perfect 3-D crystalline phase.

The XRD patterns collected on samples at different times during the enzymatic degradation at 50°C do not present prominent variations as shown in Supplementary Figure S14, in agreement with DSC data. The profiles do not change in shape and peak area, with respect to the bell-shaped halo due to amorphous component. Accordingly, we can state the process does not induce any change in crystal structure and the variations of crystallinity index at the longer times are within the experimental errors. The profile intensities of 96 h samples are significantly weak compared to the control, confirming a strong sample depletion. Patterns of the samples incubated at 65°C show analogous results.

In conclusion, on the basis of the obtained results, two scenarios could be plausible: 1) enzymatic degradation simultaneously occurred at crystalline and non-crystalline regions and 2) degradation occurs preferentially at the amorphous regions, but the extensive surface degradation of amorphous phase determined the detachment of the crystalline phase of the outermost layer.

## 4 Conclusion

This work explores for the first time the enzymatic hydrolysis of 5- and 6-carbon-containing diol subunit polymers such as PPeTF and PHTF, while investigates the semicrystalline PBTF (containing 4-carbon atoms diol), the data previously published regarding on the contrary amorphous PBTF film.

As concerning the degradation of polyester biofilms, Thc_cut1 and Thc_cut2 exhibit better activity at high temperatures (65°C vs. 50°C), with Thc_cut1 being more active than Thc_cut2. Both enzymes show an endo-wise activity, which can likely be exerted randomly along the polymer backbone. It is known that the endo-wise activity is not only due to the characteristics of the enzyme but also due to the characteristics of the polymer, including the characteristics of an ordered phase; mainly, packing efficiency, crystallinity degree, and polymer inter-chain interactions. Weak interchain interactions may favor the random cleavage of ester bonds still enclosed into the polymer film, thus not necessarily implying exo-wise activity to degrade a polymeric matrix. Probably due to the synergistic combination of these properties, related to the enzyme and to the material, it was possible to achieve the complete, or almost complete, polymer degradation within 72 h, as in the case of PPeTF and PHTF. Although the studied polyesters have a very similar chemical structure, the length of the diol chain turns out to impart very different degradability features, with PPeTF and PHTF exhibiting a much faster degradability profile than that of PBTF. Our study draws attention on PPeTF and PHTF and candidates them as excellent biobased and biodegradable materials for replacing oil-based plastics, especially in the field of packaging and thin-film applications.

## Data Availability

The original contributions presented in the study are included in the article/Supplementary Material, and further inquiries can be directed to the corresponding authors.
