# Supplementary material for: Unveiling the Enzymatic Degradation Process of Biobased Thiophene Polyesters
Source: Front Chem. 2021 Nov 15;9:771612. doi: 10.3389/fchem.2021.771612 (PMC8634338; doi:10.3389/fchem.2021.771612)
Supplement: Supplementary file 1 [file DataSheet1.docx]

**Electronic Supplementary Information**

Unveiling the enzymatic degradation process of bio-based thiophene polyesters

Federico A. Bertolini^a^, Michelina Soccio^b^, Simone Weinberger^a^, Giulia Guidotti^b^ Massimo Gazzano^c^, Georg M. Guebitz^a,d^, Nadia Lotti^b,*^, and Alessandro Pellis^a,d,e*^

^a^University of Natural Resources and Life Sciences, Vienna, Department of Agrobiotechnology, Institute of Environmental Biotechnology, Konrad Lorenz Strasse 20, 3430, Tulln an der Donau, Austria

^b^University of Bologna, Department of Civil, Chemical, Environmental and Materials Engineering, Via Terracini 28, 40131 Bologna, Italy

^c^Organic Synthesis and Photoreactivity Institute, CNR, Via Gobetti 101, 40129, Bologna, Italy

^d^Austrian Centre of Industrial Biotechnology, Konrad Lorenz Strasse 20, 3430, Tulln an der Donau, Austria

^e^University of Natural Resources and Life Sciences, Vienna, Core Facility Bioactive Molecules Screening and Analysis, Konrad Lorenz Strasse 20, 3430, Tulln an der Donau, Austria

*Correspondence to: Dr. Alessandro Pellis alessandro.pellis@boku.ac.at and Prof. Nadia Lotti nadia.lotti@unibo.it

*
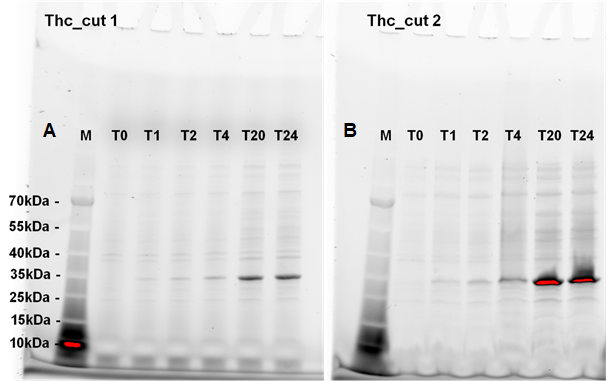
***Figure S1.** Expression analysis of Thc_cut1 (A) and Thc_cut2 (B). SDS-PAGE was carried out on crude extracts of *E. coli* cells withdrawn at different times after induction (T0-T24).

*
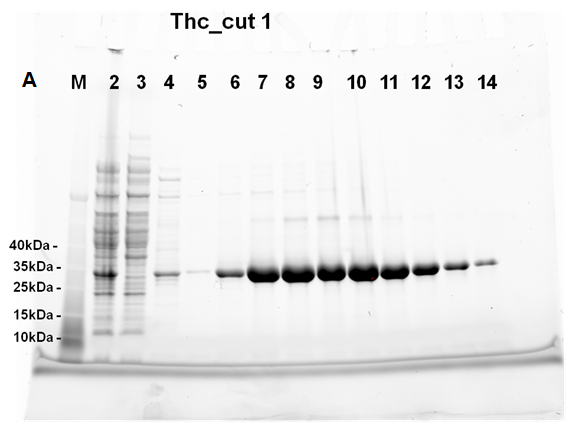
*

*
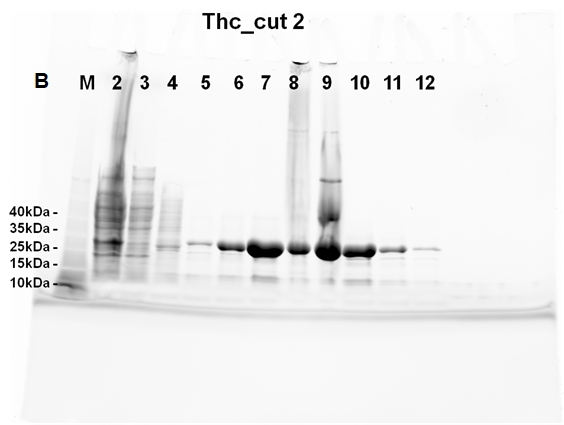
*

**Figure S2.** SDS-PAGE analysis of IMAC purifications. (A) Thc_cut1: 1, protein marker; 2, crude extract; 3-4, flow through; 5-14, elution fractions. (B) Thc_cut2: 1, protein marker; 2, crude extract; 3, flow through 4-12, elution fractions.


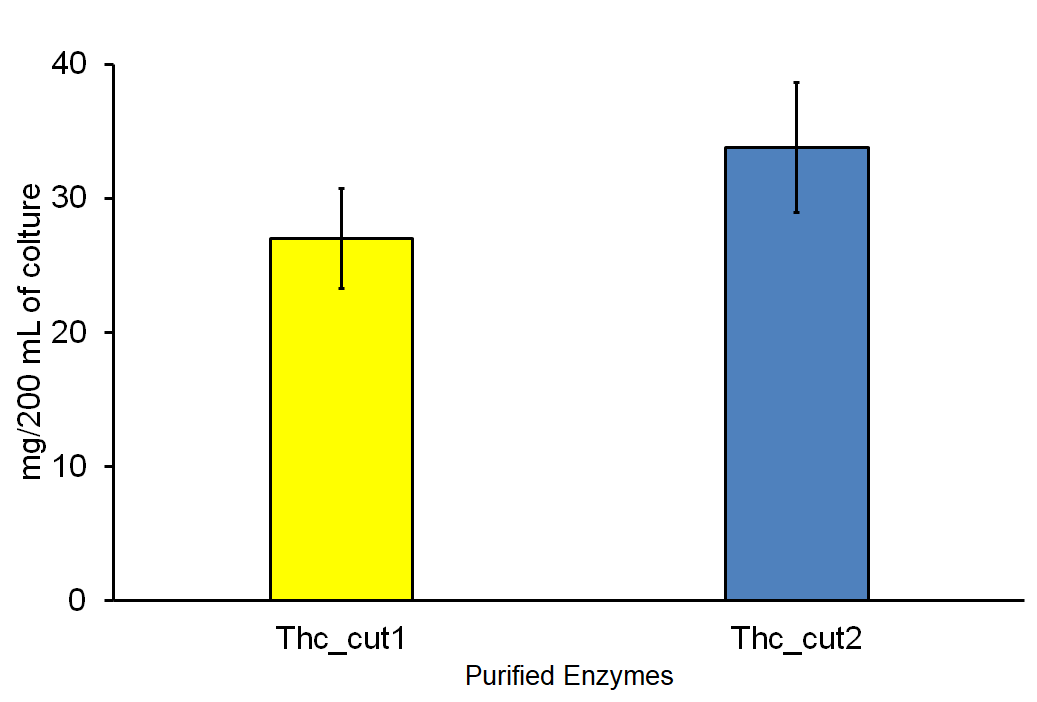


**Figure S3.** Purification yield for Thc_cut1 (yellow, left) and Thc_cut2 (blue, right). The experiments were performed in triplicate and the average values ± standard deviations are shown.


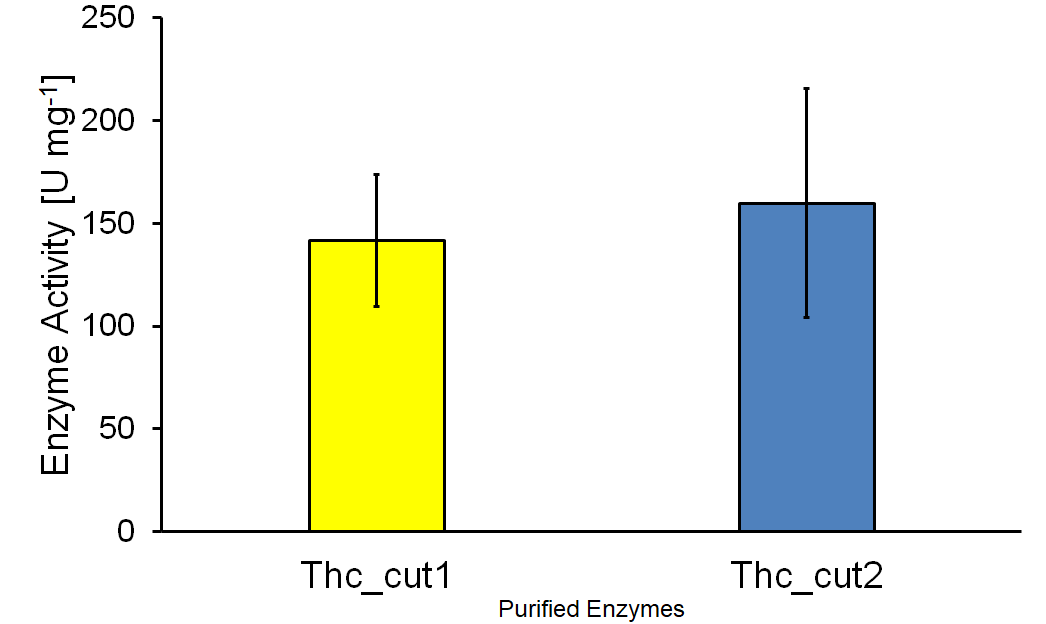


**Figure S4.** Hydrolytic activity of Thc_cut1 (yellow bar) and Thc_cut2 (blue bar) on pNPB. The experiments were performed in triplicate and the average values ± standard deviations are shown.


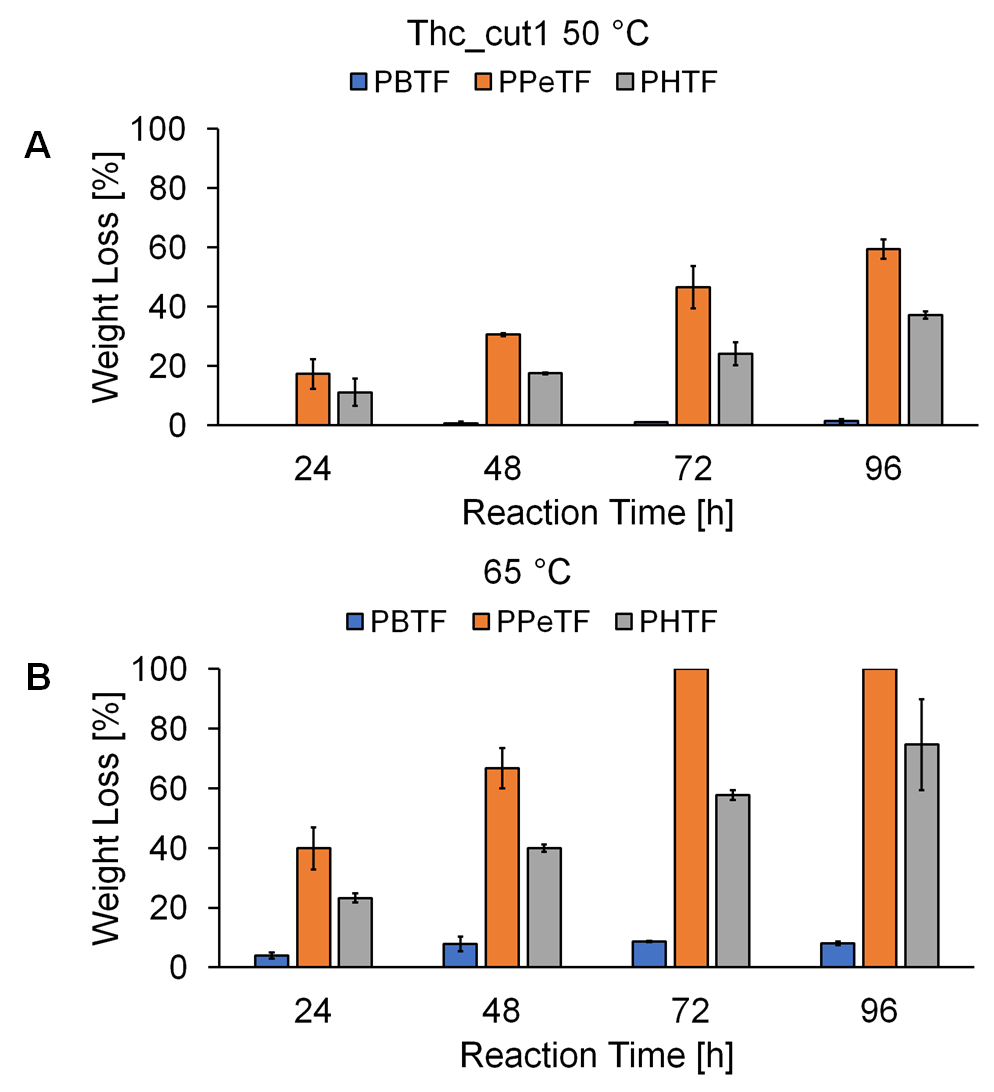


**Figure S5.** HPLC analysis from the enzymatic hydrolysis of TPCA-based polyesters using Thc_cut1 at 50 °C. All experiments were performed in quadruplicate and the average values ± the standard deviations are shown.


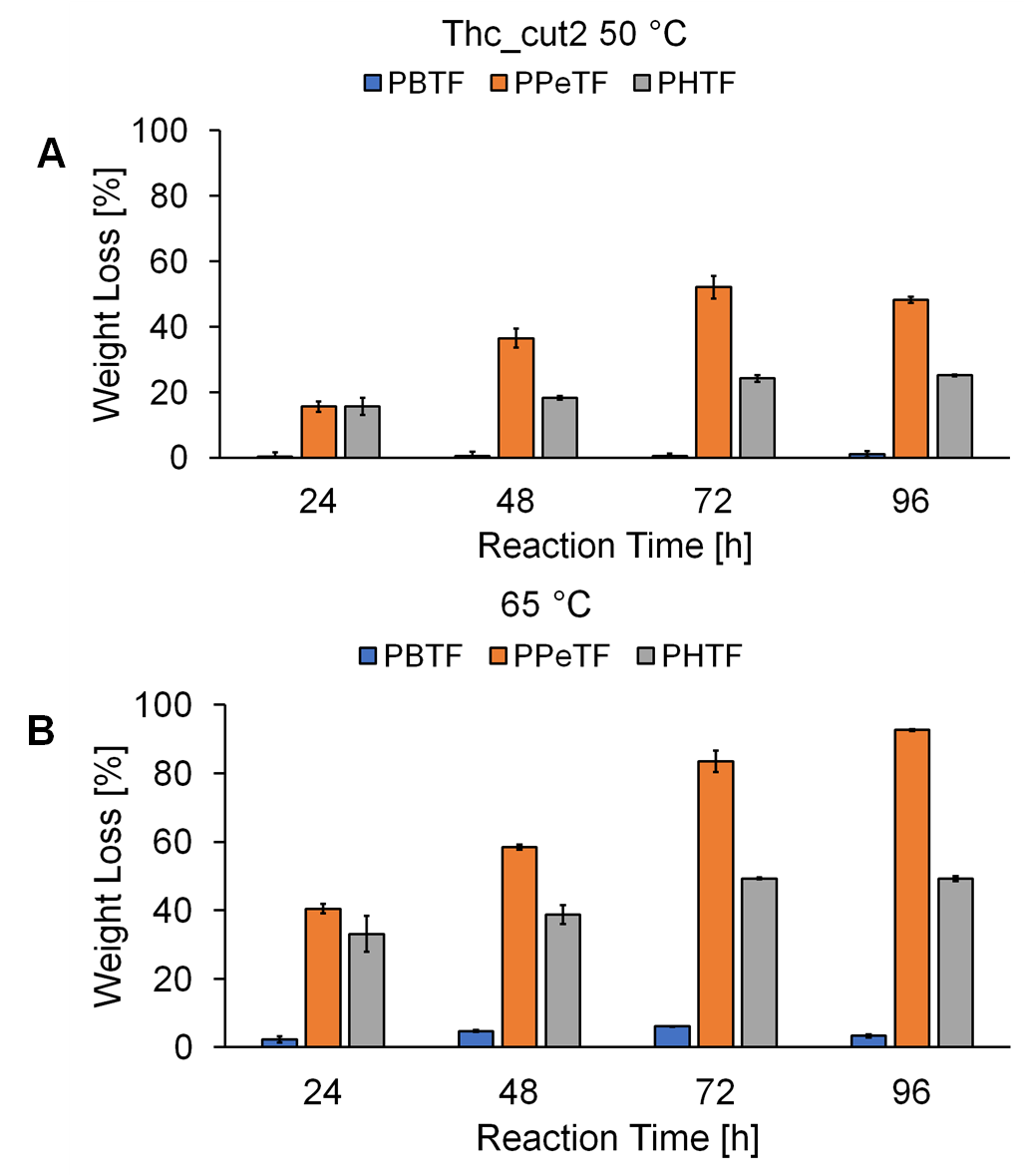


**Figure S6.** HPLC analysis from the enzymatic hydrolysis of TPCA-based polyesters using Thc_cut2 at 50 °C. All experiments were performed in quadruplicate and the average values ± the standard deviations are shown.


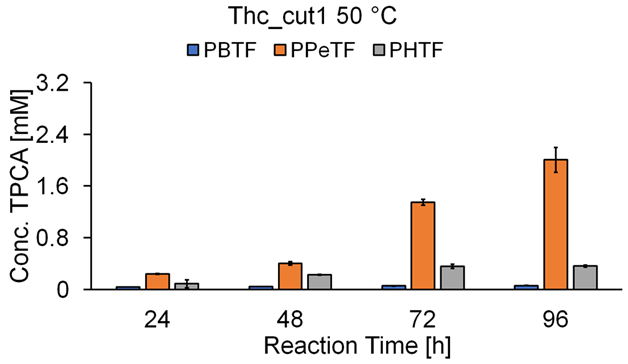


**Figure S7.** HPLC analysis from the enzymatic hydrolysis of TPCA-based polyesters using Thc_cut1 at 50 °C. All experiments were performed in quadruplicate and the average values ± the standard deviations are shown.


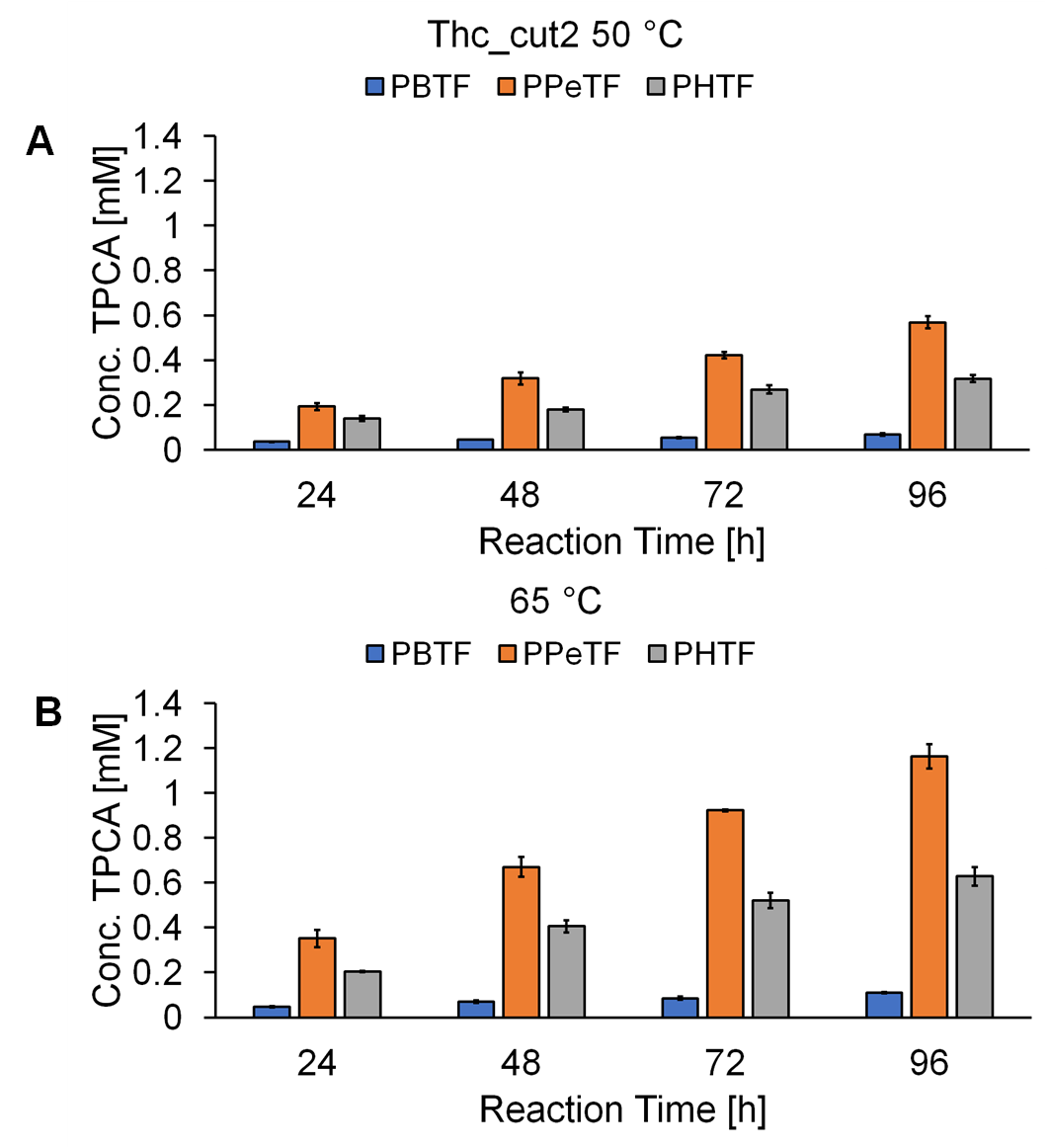


**Figure S8.** HPLC analysis from the enzymatic hydrolysis of TPCA-based polyesters using Thc_cut2 at 50 °C. All experiments were performed in quadruplicate and the average values ± the standard deviations are shown.


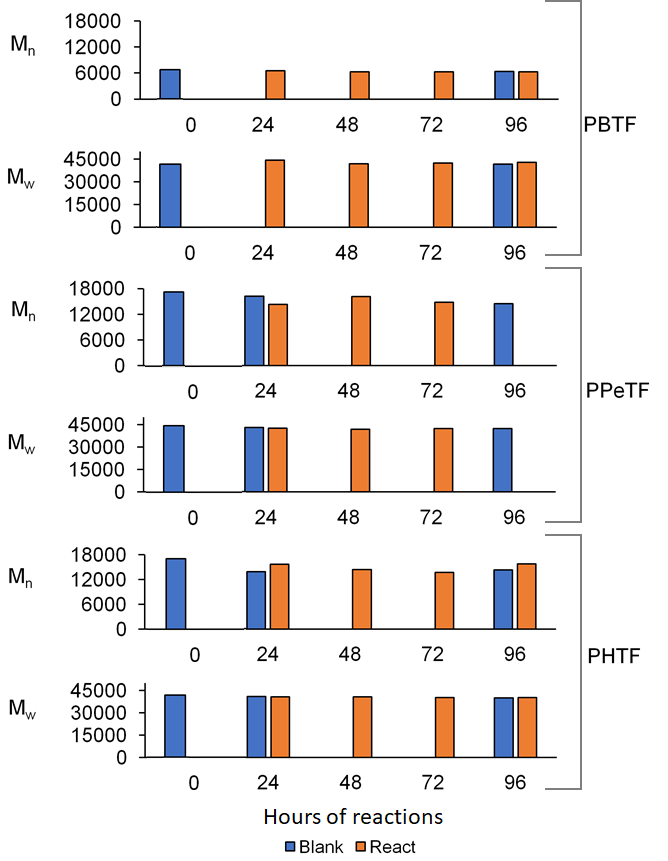


**Figure S9.** Gel permeation chromatography carried out on TPCA-based polyester films treated over time with Thc_cut1 at 65°C. Blue bars represent the untreated controls (incubated in the saline buffer without enzyme), orange bars represent the samples subjected to hydrolysis. The data are presented in terms of M_w_ and M_n_.


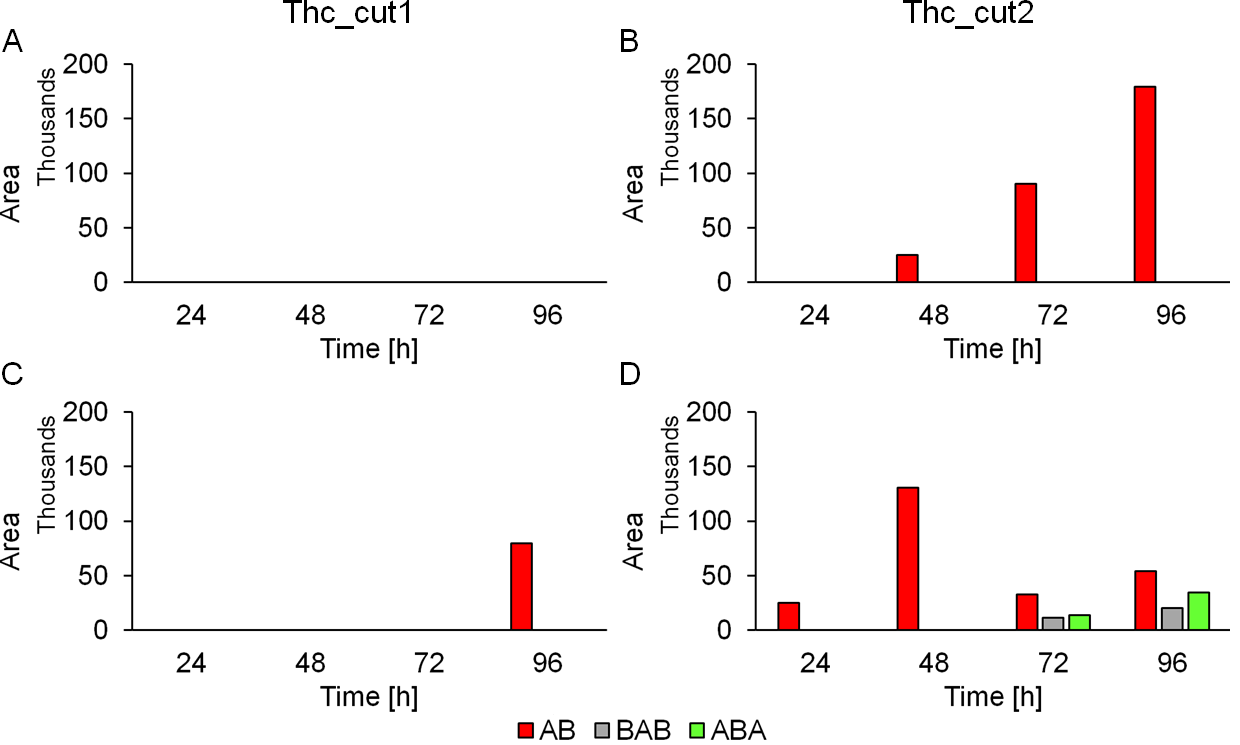


**Figure S10.** Released oligomers determined via HPLC-TOF/MS of PHTF polymer. Time course reaction showing the released dimers AB, ABA and BAB for the enzymatic hydrolysis performed at 50°C (panels A and B) and 65°C (panels C and D) with Thc_cut1 and Thc_cut2. The reactions were performed in 1 M KH2PO4/K2HPO4 buffer pH 8.0. No released products were detected in the control reactions.


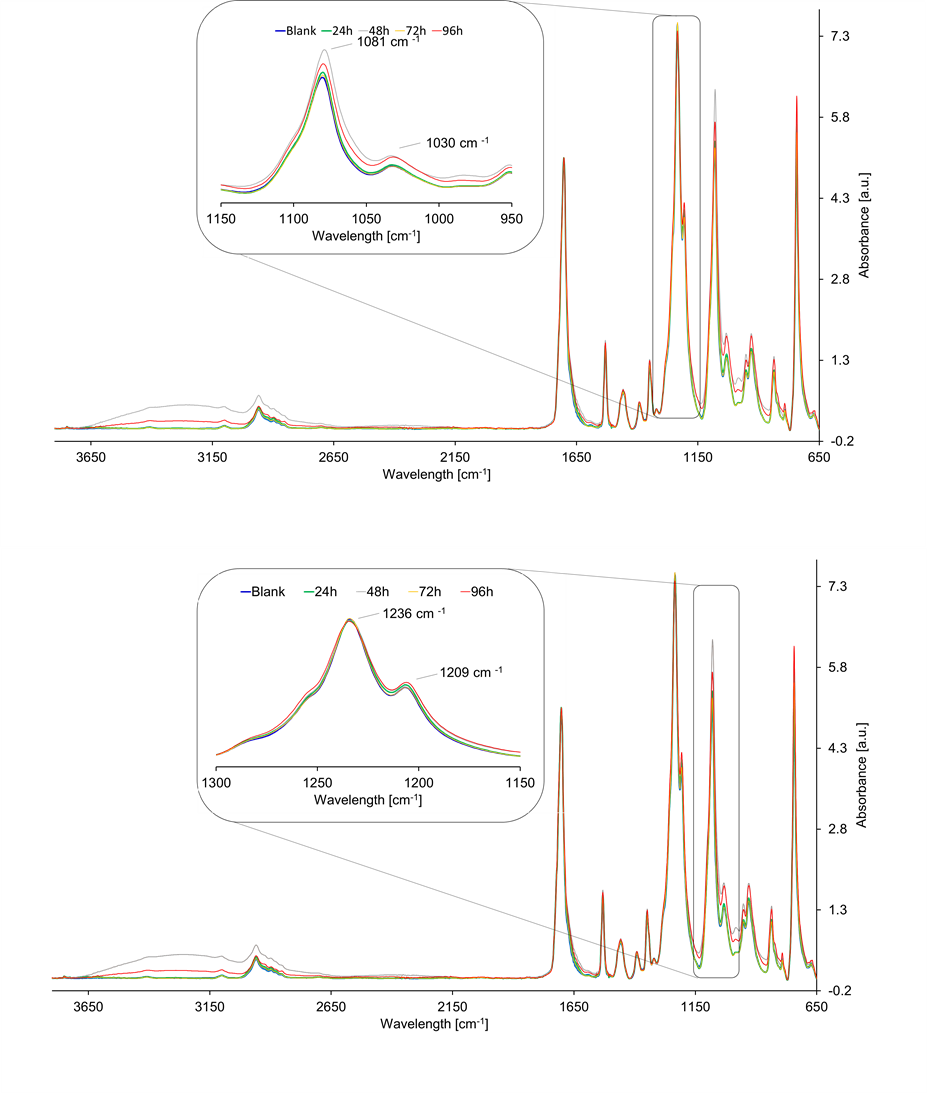


**Figure S11**. FT-IR spectra of partially hydrolyzed PBTF films. Analyzed samples were treated with Thc_cut1 at 65°C, for 96 h. The spectra were normalized with respect to the intensity of the 1703 cm^-1^ peak.


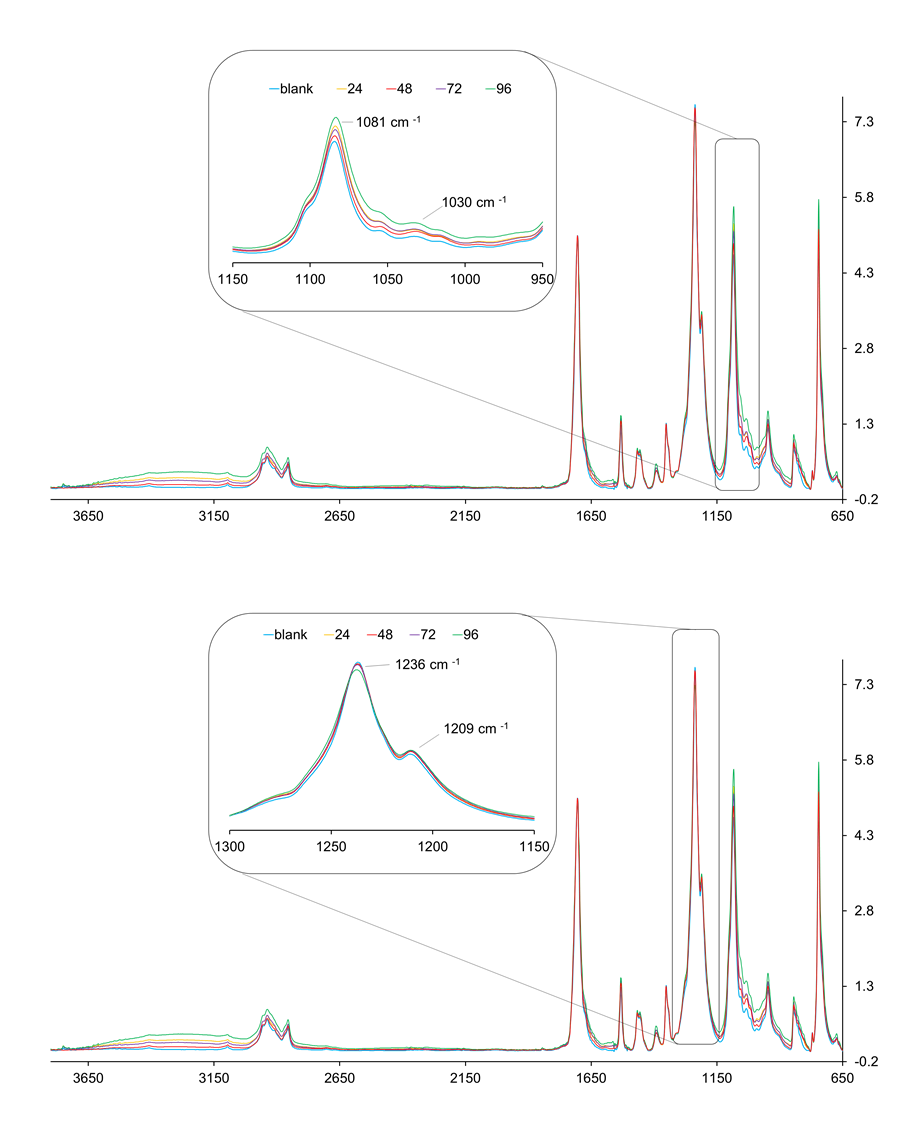


**Figure S12***.* FT-IR spectra of partially hydrolyzed PHTF films. Analyzed samples were treated with Thc_cut1, at 65°C for 96 h. The spectra were normalized with respect to the intensity of the 1703 cm^-1^ peak.

| **Table S1.** HPLC method used for TPCA detection and quantification. | | |
| --- | --- | --- |
| Time  [min] | A [MeOH]  [%] | B [HCOOH]  [%] |
| 0 | 30 | 70 |
| 2 | 50 | 50 |
| 9 | 100 | 0 |
| 18 |  |  |
| 19 | 30 | 70 |
| 20 |  |  |

| **Table S2*.*** LC-TOF/MS method used for TPCA soluble oligomers detection. | | |
| --- | --- | --- |
| Time  [min] | A  [20 mM NH_4_COOH] [%] | B  [AcN] [%] |
| 0 | 100 | 0 |
| 1 |  |  |
| 11 | 0 | 100 |
| 12 |  |  |
| 14 | 100 | 0 |
| 15 |  |  |

| **Table S3.** Thermal characterization data (T_m_ and ΔH_m_) of the partially degraded samples and their blanks. | | | | | | | | | | | | | | | | | | | |
| --- | --- | --- | --- | --- | --- | --- | --- | --- | --- | --- | --- | --- | --- | --- | --- | --- | --- | --- | --- |
| **PPeTF** |  | **50 °C** | | | | | | | | **65 °C** | | | | | | | | | |
|  | **Enzyme** | **Thc_cut1** | | | | **Thc_cut2** | | | | **Thc_cut1** | | | | **Thc_cut2** | | | | | |
|  | **Time (h)** | **24** | **48** | **72** | **96** | **24** | **48** | **72** | **96** | **24** | **48** | **72** | **96** | **24** | | **48** | | **72** | **96** |
| *blank* | **T_m_ (°C)** | 65 | 66 | 66 | 66 | 65 | 66 | 66 | 66 | 72 | 72 | - | - | | 72 | | 72 | 73 | - |
|  | **ΔH_m_ (J/g)** | 25 | 26 | 28 | 27 | 26 | 26 | 26 | 27 | 29 | 29 | - | - | | 27 | | 29 | 28 | - |
| *degraded* | **T_m_ (°C)** | 65 | 66 | 66 | 67 | 65 | 66 | 67 | 66 | 72 | 73 | - | - | | 73 | | 73 | 72 | - |
|  | **ΔH_m_ (J/g)** | 26 | 26 | 29 | 28 | 25 | 26 | 26 | 26 | 29 | 27 | - | - | | 28 | | 27 | 29 | - |

| **PHTF** |  | **50 °C** | | | | | | | | **65 °C** | | | | | | | |
| --- | --- | --- | --- | --- | --- | --- | --- | --- | --- | --- | --- | --- | --- | --- | --- | --- | --- |
|  | **Enzyme** | **Thc_cut1** | | | | **Thc_cut2** | | | | **Thc_cut1** | | | | **Thc_cut2** | | | |
|  | **Time (h)** | **24** | **48** | **72** | **96** | **24** | **48** | **72** | **96** | **24** | **48** | **72** | **96** | **24** | **48** | **72** | **96** |
| *blank* | **T_m_^I^ (°C)** | 63 | 64 | 65 | 64 | 63 | 64 | 63 | 64 | 72 | 73 | 73 | 73 | 72 | 72 | 73 | 73 |
|  | **ΔH_m_^I^ (J/g)** | 10 | 10 | 11 | 11 | 9 | 10 | 10 | 12 | 16 | 18 | 16 | 16 | 15 | 16 | 16 | 16 |
|  | **T_m_^II^ (°C)** | 95 | 95 | 95 | 95 | 95 | 95 | 95 | 95 | 96 | 95 | 95 | 95 | 95 | 95 | 95 | 95 |
|  | **ΔH_m_^II^ (J/g)** | 25 | 24 | 24 | 27 | 23 | 22 | 27 | 26 | 21 | 24 | 21 | 19 | 21 | 22 | 19 | 21 |
| *degraded* | **T_m_^I^ (°C)** | 63 | 64 | 64 | 64 | 64 | 64 | 65 | 65 | 72 | 73 | 73 | 73 | 72 | 72 | 73 | 73 |
|  | **ΔH_m_^I^ (J/g)** | 10 | 10 | 11 | 11 | 9 | 10 | 10 | 12 | 15 | 17 | 16 | 16 | 15 | 17 | 16 | 17 |
|  | **T_m_^II^ (°C)** | 95 | 95 | 95 | 95 | 95 | 95 | 95 | 95 | 96 | 95 | 95 | 95 | 95 | 95 | 95 | 95 |
|  | **ΔH_m_^II^ (J/g)** | 26 | 24 | 25 | 25 | 24 | 21 | 26 | 26 | 20 | 23 | 21 | 19 | 21 | 23 | 19 | 21 |

**Figure S13.** DSC curves of the samples at different degradation times and their relative blanks: A-D) PPeTF; E-H) PHTF.

**Figure S14.** XRD patterns of the samples after enzymatic degradation at 50°C. Peak intensity of patterns of samples after 96 h of enzyme digestion are multiplied by two. Dotted and dashed line are reported to highline crystalline and amorphous components for sample Thc_cut2_96h, as an example.
